# Burden and geographic distribution of oral cavity and oropharyngeal cancers in the Russian Federation

**DOI:** 10.3389/fonc.2023.1197287

**Published:** 2023-08-03

**Authors:** Anastasiya Muntyanu, Vladimir Nechaev, Elena Pastukhova, James Logan, Elham Rahme, Andrei Zubarev, Elena Netchiporouk, Ivan V. Litvinov

**Affiliations:** ^1^ Department of Experimental Medicine, McGill University, Montreal, QC, Canada; ^2^ Division of Dermatology, McGill University, Montreal, QC, Canada; ^3^ Faculty of Medicine, University of Ottawa, Ottawa, ON, Canada; ^4^ Geographic Information System (GIS), Ottawa, ON, Canada; ^5^ Division of Clinical Epidemiology, McGill University, Montréal, QC, Canada

**Keywords:** squamous cell carcinoma, oral cavity cancer, pharyngeal cancer, human papilloma virus (HPV), epidemiology, incidence, mortality, Russian Federation

## Abstract

**Background:**

The global incidence of lip and oral cavity cancers (OCCs) and oropharyngeal cancers (OPCs) is steadily increasing. While tobacco and alcohol consumption are established risk factors, a considerable proportion of these cancers has become attributed to human papilloma virus (HPV) infection. We aimed to describe the occurrence and identify potential risk factors of OCCs and OPCs across the Russian Federation during 2007-2018.

**Methods:**

We conducted an ecological analysis using publicly accessible data from the P.A. Herzen Moscow Oncology Research Institute. Incidence and mortality rates by jurisdiction were mapped for geospatial analysis. We pre-defined 11 potential contributing risk factors and used univariable and multivariable Poisson regression model with backwards stepwise variable selection to identify associated factors with OCC and OPC.

**Results:**

A total of 190,585 individuals were diagnosed with OCCs and OPCs in Russia between 2007-2018. Non-uniform geographic distribution of cancer cases was noted where the Far Eastern Federal District had the highest rate of OCC and the Central Federal District of OPCs. Districts with high weekly alcohol consumption had significantly higher incidence and mortality rates in both sexes. Districts with high rates of daily smoking had higher incidence of OCC among females, and those with low smoking trends had lower mortality rates for OCCs and OPCs.

**Conclusion:**

We detail the burden of OCCs and OPCs across Russia, with the aim of elucidating modifiable risk factors and proposing evidence-based prevention strategies. Tobacco/alcohol sales control measures and smoking/drinking cessation programs should continue to be prioritized as public health measures, especially for females.

## Introduction

Lip and oral cavity cancers (OCCs) and oropharyngeal cancers (OPCs) carry a significant burden of morbidity and mortality, with estimated 614,000 incident cases worldwide in 2020 (1, 2). In the Russian Federation, these cancers accounted for approximately 3.6% of all cancer-related deaths based on the 2020 GLOBOCAN data (2). Squamous cell carcinoma (SCC) makes up approximately 90% of OCCs and OPCs (3, 4). Tobacco and alcohol consumption are established risk factors for both OCC and OPC often, conferring a synergistic effect (5). Additionally, exposure to environmental pollutants (*i.e.*, formaldehyde, wood dust) (6), immunosuppression, Epstein Barr virus infection, betel nut chewing, and lower socioeconomic status (SES) have also been implicated in carcinogenesis (3, 7). Cumulative ultraviolet radiation (UVR) exposure is an established risk factor in lip SCC, a subtype of OCC (8). Importantly, human papilloma virus (HPV) infection has been strongly associated with OPC, while its role in OCC carcinogenesis remains less evident (9, 10).

The objective of this national populational-based study was to comprehensively describe incidence and mortality rates by geographical region over the years 2007-2018 and determine associated risk factors in the Russian Federation, a wide, diverse, and multi-national region of the world. Our findings could help direct public health prevention strategies including smoking cessation, reduction in alcohol consumption, as well as HPV vaccination and education programs.

## Methods

### Study design

We conducted an ecological analysis with methods similar to previously published research (11, 12). Study design and data reporting were performed in accordance with Strengthening the Reporting of Observational Studies in Epidemiology (STROBE) checklist (13).

### Setting/participants/data sources

The incidence and, where possible, mortality data were extracted for OCC (C00-C09), and pharyngeal cancers (C10-C13) with other unspecified sites in the lip, oral cavity and pharynx (C14) from the publicly available annual reports of the P.A.Herzen Moscow Oncology Research Institute, Ministry of Health of Russian Federation, based on International Classification of Diseases Version 10(ICD-10) as listed in parentheses (14, 15).

The P.A. Herzen Moscow Oncology Research Institute collects and analyzes the data based on ICD codes received from primary regional oncology centers/hospitals in compliance with uniform regulations from Russian Ministry of Health. Together, these regional registries provide cancer data (based on pathological diagnosis) for the entire population, including populations in remote and sparsely populated territories. The local organizations collect primary data (individual level data) and provide annual reports to higher level authorities at the national level (aggregated and depersonalized data accessible for study). The registry system undergoes data quality control procedures at all stages of information gathering to verify for accuracy and ensure there are no duplications. Cancer reporting standards in Russia are aligned with the stringent regulation established by the International Agency for Research on Cancer (IARC).

The raw populational cancer data of interest, subdivided by age group, sex, and jurisdiction were available for 2007-2018 years. Hence, this time interval was chosen for the study.

The Russian Federation consists of eight Federal Districts that are conglomerates of Federal Subjects (*i.e.*, individual jurisdictions). Federal Subjects include oblast, province, krai, autonomous republic, autonomous oblast, and cities with a special status (*i.e.*, Moscow and Saint Petersburg). Population data by region, by age, and sex were obtained from the Federal State Statistics Service (Rosstat) for 2008-2009, and 2011-2018. Data for 2007 was not available and this year was excluded for the age-standardized rate calculation by region. Data for 2010 was obtained from the Russian Federation Population Census (Rosstat). The translated names of administrative territories were obtained from the US Embassy to Russia, which may differ from the Russian State Standard (GOST7.67-2003) (16).

### Risk factors (variables)/data sources

Details on risk factors studied (smoking, alcohol, sexually transmitted infections (STIs), and tuberculosis (TB)) and their data sources are attached in **Supplementary Table 1**. TB infection rates were included as they are typically associated with low-to-middle income communities where there may be poor access to healthcare, undernutrition, alcohol use disorder, tobacco smoking, and co-infection with HIV (17–19).

### Statistical methods

Statistical methodology is similar to our previous study on cervical cancer in the Russian Federation (12). Incidence data was reported and analyzed in several categories: OCC (C00-C09), OPC (C10-C13), and combined lip, oral and pharyngeal cancer ‘OCPCs’ (C00-C14). Mortality data for OCC and OPC were grouped in their original source ‘OCPCs’ (C00-C14). Age-standardized incidence and mortality rates (ASIRs and ASMRs, respectively) for 2008-2018, of OCCs, OPCs, and OCPCs for each Federal District and Federal Subject were calculated using the regional age population distribution from the Rosstat Census (20). For annual data on the Russian Federation as a whole, age-standardization for 2007-2018 was done using the World Health Organization (WHO) World 2000-2025 Population Standard to allow comparison with global literature. To determine trends in ASIRs and ASMRs over time, linear regression was performed as previously described (12).

To analyze the relationships between risk factors for both sexes combined as well as separately, in addition to ASIRs and ASMRs (Russian Population Standard) of OCCs, OPCs, and OCPCs, univariate and multivariate Poisson regression model analyses were used, with the jurisdiction as the unit of the analysis. A backwards stepwise variable selection by Akaike Information Criterion (AIC) was performed using a predetermined list of 11 variables of interest to identify the most important risk factors. Regions with missing data were excluded. We further checked for collinearity among predictors by assessing overall and individual variance inflation factors (VIF) with the mctest package in R version 4.0.4 for the model with the lowest AIC. Data analysis was performed using R Studio and SAS9.4 software. Where applicable, p<0.05 was accepted as significant.

Geographic maps of ASIR/ASMR across the Russian Federation were generated as previously described (12).

## Results

### Participants/descriptive data

The population of the Russian Federation is approximately 145 million individuals. On average, annually there were 10,992 patients diagnosed with OCCs between 2007-2018 (females 28.3%, males 71.7%) and 4,891 patients diagnosed with OPCs (females 14.2%, males 85.8%) (**Supplementary Tables 2-4**). On average, there were 9,329 deaths per year (females 19.3%, males 80.7%) due to OCPCs (**Supplementary Table 5**). Lip cancer patients accounted for 20%-35% of the cohort between 2007-2018. (i.e., 35% in 2007 and 20% in 2018). Analysis based on different age groups revealed that 82.1% of patients with OCCs were >50 years of age (78.1% females and 83.7%males) (**Supplementary Table 2**). Most males were diagnosed with OCCs between the ages 50-69, while for females, the diagnosis per decade remained nearly stable between 50-79 years of age. OPC age trends were similar for both sexes (**Supplementary Table 3**).

### Outcome data (incidence and mortality)

The average annual crude and ASIR of OCCs (C00-C09) for both sexes were 7.63/100,000 (95%CI 7.45-7.81) and 5.23/100,000 (95%CI 5.16-5.30), respectively (**Supplementary Table 6**). The average crude and ASIR of OPCs (C10-C13) for both sexes were 3.39/100,000 (95%CI 3.19-3.60) and 2.40/100,000 (95%CI 2.31-2.48), respectively (**Supplementary Table 7**). The average crude and ASIR of OCPCs (C00-C14) for both sexes were 11.03/100,000 (95% CI 10.64-11.41) and 7.62/100,000 (95% CI 6.49-7.76) and mortality rates were 6.48/100,000 (95%CI 6.27-6.68) and 4.44/100,000 (95%CI 4.38-4.50), respectively (**Supplementary Tables 8, 9**). In comparison to ASIR estimates of countries around the world (based on data from International Agency for Research of Cancer) in 2012, Russian Federation was found to rank 10^th^ in terms of ASIR for males and 27^th^ for females (**Supplementary Table 10**).

### Main results

**Figure 1:**
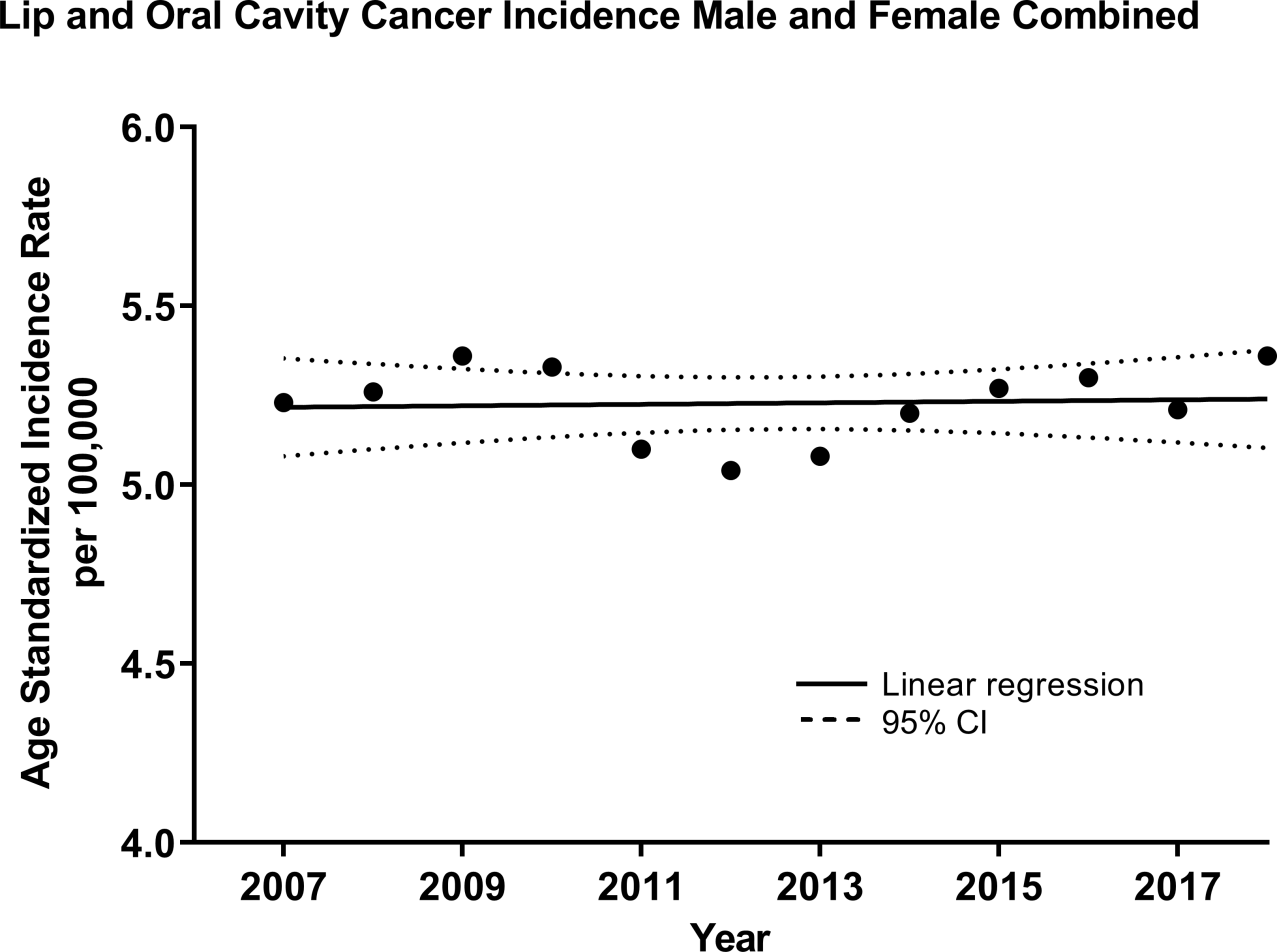
Age-standardized (WHO World Population Standard 2000-2025) incidence of lip and oral cavity cancers (OCCs) (C00-C09) for both sexes combined in the Russian Federation, 2007-2018. Each data point indicates the age-standardized incidence rate for a given year. The solid line represents the line of best fit, and dotted lines indicate the corresponding upper and lower 95% Confidence Intervals (CI). Coefficient of determination is represented by R^2^. Statistical significance is expressed in p-values. Slope of the line is 0.0021/100,000 individuals per year, R^2 = ^0.005, p=0.8285.

Linear regression analysis of the ASIR revealed that OCC incidence increased by 0.0021/100,000 individuals per year (R^2 = ^0.005, *p*=0.8285, **Figure 1**) for both sexes, and the trend appeared stable. However, stratification by sex showed a decreasing trend for males –0.075/100,000 (R^2 = ^0.63, p=0.002, **Supplementary Figure 1**) and an increasing one for females 0.036/100,000 (R^2 = ^0.65, p=0.0015, **Supplementary Figure 2**). As a result, the male-to-female incidence rate ratio (IRR) was decreasing over time with a slope of -0.088 (R^2 = ^0.85, p<0.0001, **Supplementary Figure 3**).

**Figure 2:**
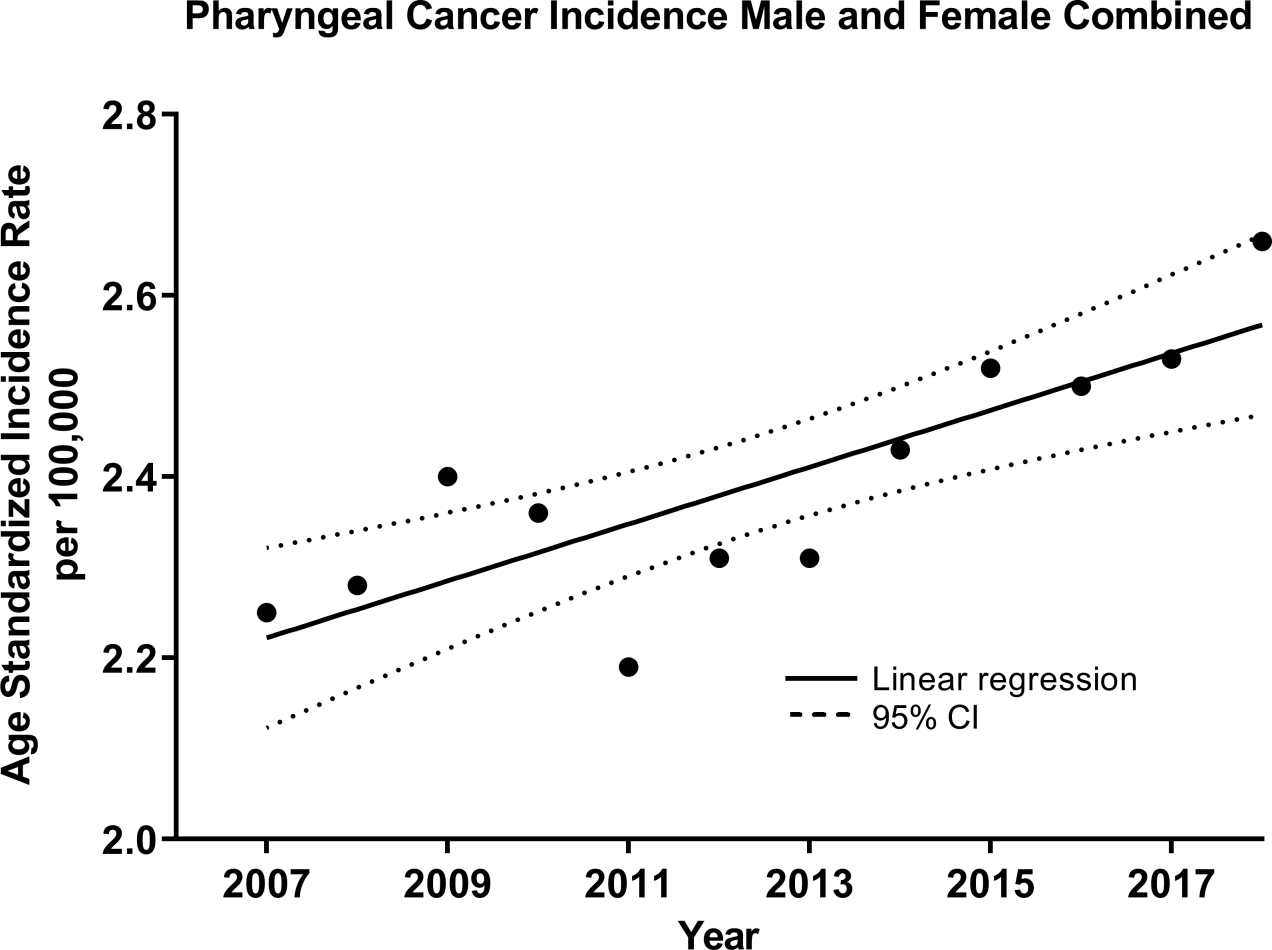
Age-standardized Incidence (WHO World Population Standard 2000-2025) of oropharyngeal cancers (OPCs) (C10-C13) for both sexes combined in the Russian Federation, 2007-2018. Each data point indicates the age-standardized incidence rate for a given year. The solid line represents the line of best fit, and dotted lines indicate the corresponding upper and lower 95% Confidence Intervals (CI). Coefficient of determination is represented by R^2^. Statistical significance is expressed in p-values. Slope of the line is 0.0314/100,000 individuals per year, R^2 = ^0.68, p=0.001.

For OPCs (C10-C13), an increasing trend for both sexes was observed over time at a rate of 0.031 cases per 100,000 individuals per year (R^2 = ^0.68, p=0.001, **Figure 2**). Similarly, the trend was increasing for males at a rate of 0.048 (R^2 = ^0.53, p=0.0069, **Supplementary Figure 4**) and for females, although at a slower rate, 0.014 (R^2 = ^0.67, p=0.0011, **Supplementary Figure 5**). Thus, the male-to-female IRR was decreasing -0.10 over time (R^2 = ^0.58, p=0.0038, **Supplementary Figure 6**).

Mortality was presented for OCPCs (C00-C14). For both sexes combined, there was a non-significant upward trend over time 0.013 deaths per 100,000 individuals per year (R^2 = ^0.21, p=0.13, **Supplementary Figure 7**). When stratified by sex, there was a decreasing trend for males -0.024 (R^2 = ^0.17, p=0.19, **Supplementary Figure 8**) and an increasing one for females 0.023 (R^2 = ^0.77, p=0.047, **Supplementary Figure 9**). Male-to-female mortality rate ratio (MRR) slope was -0.12 (R^2 = ^0.84, p<0.0001, **Supplementary Figure 10**).

### Geographic distribution of OCCs and OPCs

**Figure 3:**
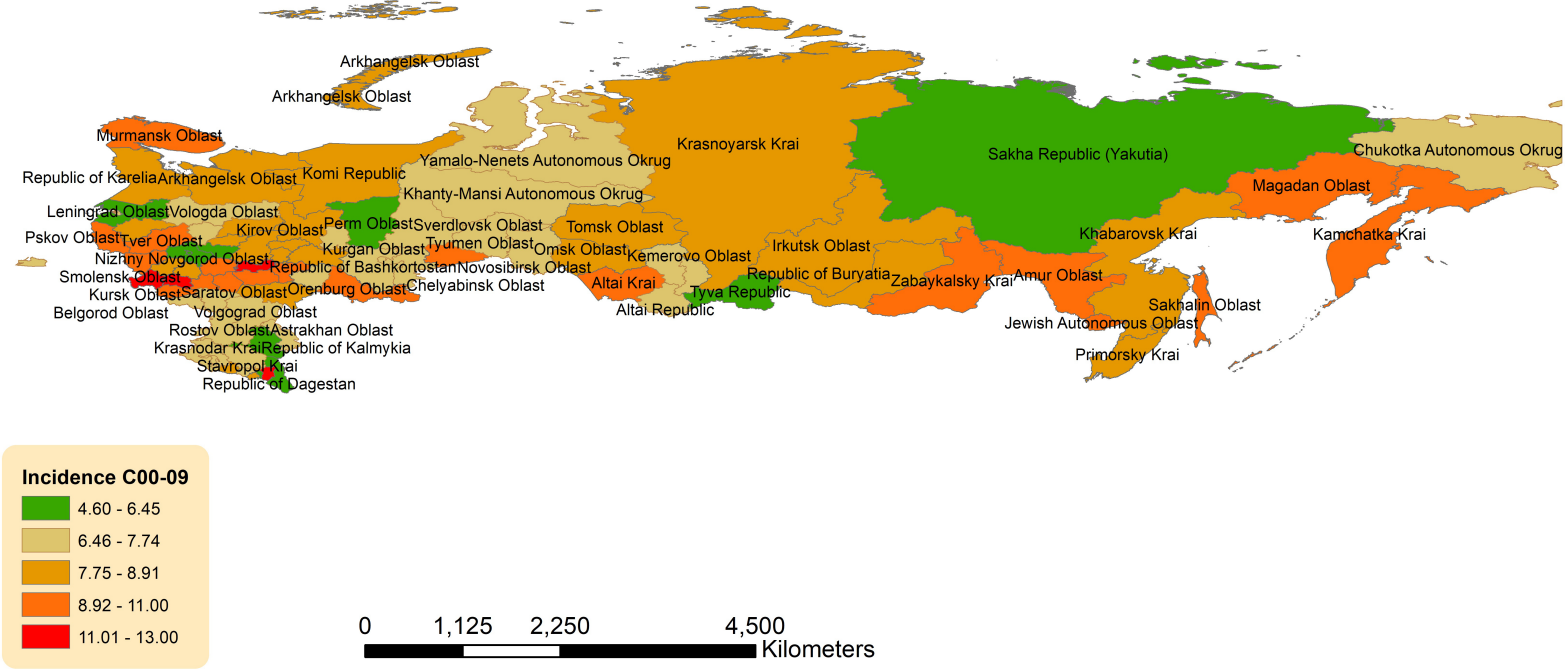
Geographic distribution of lip and oral cavity cancers (OCCs) (C00-09) average age-standardized incidence rate for both sexes combined in the Russian Federation, 2008-2018.

**Figure 4:**
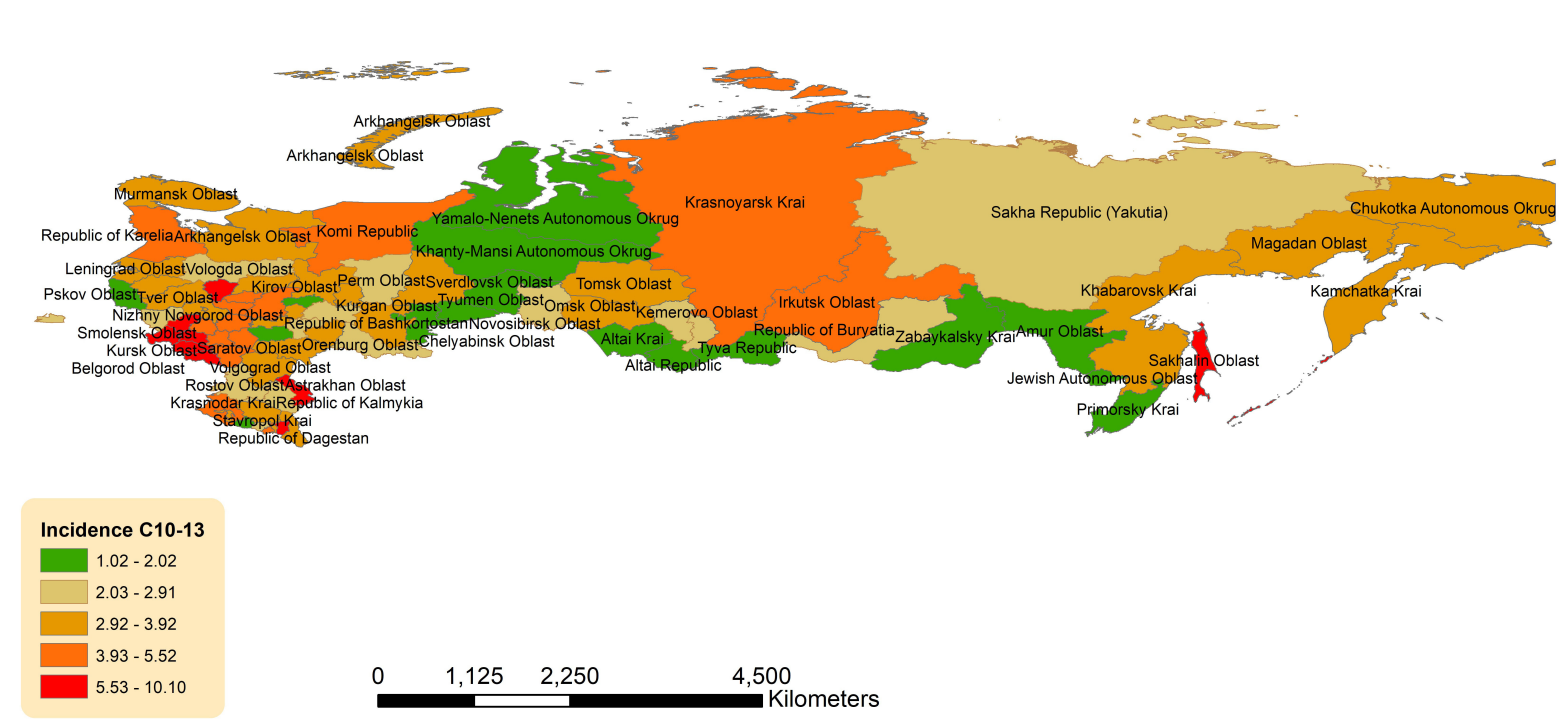
Geographic distribution of pharyngeal cancers (OPCs) (C10-13) average age-standardized incidence rate for both sexes combined in the Russian Federation, 2008-2018.

Geographic distribution across the Russian Federation for age-standardized incidence of OCCs, OPCs is presented in **Figures 3**, **4**.

There was a slight eastern predominance in incidence of OCCs (C00-C09) across districts, with the highest ASIR in Far Eastern Federal District (8.39, 95%CI 7.64-9.13) and Siberian Federal District (8.20, 95%CI 7.78-8.62), followed by Volga Federal District (8.16, 95%CI 7.84-8.48) (**Supplementary Table 11**; **Figure 3**). The lowest ASIR of OCCs was observed in North Caucasian Federal District (7.15, 95%CI 6.54-7.77). Of the individual jurisdictions, Bryansk Oblast (Central Federal District) had the highest crude incidence (13.07, 95%CI 11.13-15.00) and was 1.7 times the national average. Moscow City (Central Federal District) had the lowest ASIR (4.60, 95%CI 4.23-4.97).

Non-uniform geographic pattern of OPC ASIR was also observed with the higher rates in the western and southern parts of the country (**Figure 4**). Federal districts with the highest ASIRs were: Central Federal District (4.19, 95%CI 3.99-4.39), North Caucasian Federal District (3.63, 95%CI 3.19-4.06) and Southern Federal District (3.59, 95%CI 3.29-3.88) (**Supplementary Table 11**). The lowest ASIR was observed in the Ural Federal District (2.44, 95%CI 2.16-2.72). Of the individual jurisdictions, Chechen Republic (North Caucasian Federal District) had the highest ASIR, which was 3 times the national average (10.15, 95%CI 7.82-12.49), while Tyva (Siberian Federal District) had the lowest (1.02, 95%CI 0.00-2.46).

The geographic incidence data for OCPCs (C00-C14) and corresponding mortality rates are presented **Supplementary Figures 11, 12**, respectively.

### Assessment of risk factors

Poisson univariate regression analysis assessing the association of the predetermined risk factors and the incidence of OCCs and OPCs (C00-14) in both sexes analyzed together, demonstrated a significant positive predictive effect from the rate of weekly alcohol consumption - both infrequent (once/week) and frequent (2-3 times/week) by jurisdiction (**Table 1**). In the multivariate model, only rate of frequent weekly alcohol consumption was identified as significant. For OCC (C00-C09) incidence for both sexes combined, rate of frequent weekly alcohol consumption by jurisdiction demonstrated a positive relationship both in the univariate and multivariate models (**Supplementary Table 12**). For OPC (C10-C13) incidence for both sexes, rate of frequent weekly alcohol consumption once again demonstrated a positive relationship in the univariate regression model, while rate of daily smoking, TB infection, and syphilis infection each had a negative relationship (**Supplementary Table 12**). The multivariate model for both sexes, revealed that the rate of frequent weekly alcohol consumption continued to demonstrate a significant positive effect, while rate of TB infection by jurisdiction was again a negative predictor. Mortality risk factors for OCPCs (C00-C14) in both sexes are presented in **Table 1**.

**Table 1:** Poisson regression analysis of risk factors for lip, oral cavity, and pharyngeal cancers (C00-C14) on age-standardized incidence and mortality for both sexes in the Russian Federation, 2008-2018.

Incidence Rate C00-C14 (Univariate Model)					
Predictor	Coefficient ß	I.R.R.	CIs	S.E.	*p-*value
Weekly alcohol consumption (once per week)	0.02	1.017	1.002-1.031	0.01	0.02
Weekly alcohol consumption (2-3 times per week)	0.05	1.046	1.017-1.076	0.01	<0.005
Incidence Rate C00-C14 (Multivariate Model)					
Weekly alcohol consumption (2-3 times per week)	0.05	1.048	1.018-1.079	0.01	<0.005
Mortality Rate C00-C14 (Univariate Model)					
Any alcohol consumption	0.01	1.009	1.000-1.017	0.004	0.02
Weekly alcohol consumption (2-3 times per week)	0.05	1.052	1.013-1.091	0.02	0.007
Sales of alcohol content <9%	0.02	1.017	1.000-1.033	0.008	0.04
Mortality Rate C00-C14 (Multivariate Model)					
Weekly alcohol consumption (2-3 times per week)	0.05	1.054	1.015-1.093	0.02	0.006

Bolded parameter estimates denote a negative association. Coefficient ß indicates regression coefficient. I.R.R. denotes the incidence rate ratios. S.E. denotes standard error. CIs indicate confidence intervals. p-values <0.05 were considered significant. Only significant values are shown.

For males, Poisson univariate analysis of incidence of OCPCs (C00-C14), identified the following factors as positively predictive: rates of weekly alcohol consumption (once/week and frequent; 2-3 times/week), and sale of alcohol content <9% by jurisdiction (**Table 2**). On the other hand, rare alcohol consumption, smoking (<10 cigarettes/day), TB, syphilis and gonorrhea infections by jurisdiction had a negative association with OCC and OPC incidences in males. Multivariate models yielded rates of frequent weekly alcohol consumption and alcohol induced psychosis as significant positive predictors, while the rate of gonorrhea infection had a significant negative effect (**Table 2**). For OCC (C00-C09) incidence in males, rates of weekly alcohol consumption (once and frequent) and smoking (>1 pack/day) were associated with increased incidence, while rare alcohol consumption, smoking (<10 cigarettes/day), and gonorrhea infection had a negative effect (**Supplementary Table 13**). Multivariate models yielded only the rate of frequent weekly alcohol consumption as a positive significant factor for OCC incidence in males. Analysis of OPCs (C10-C13) in males using the univariate model identified numerous positive predictive factors: rates of weekly alcohol consumption (once and frequent), ‘never’ smoking, rare smoking, smoking (<5 cigarettes/day), and sale of alcohol content <9% by jurisdiction (**Supplementary Table 13**). The following negative predictors of OPC incidence were identified: rates of monthly alcohol consumption, daily smoking, smoking (>1 pack/day), TB, HIV, syphilis, and gonorrhea infections. Multivariate models demonstrated ‘any’ alcohol consumption, rate of frequent weekly alcohol consumption, sale of alcohol content <9%, alcohol induced psychosis and syndrome of alcohol dependence as positively predictive of OPC incidence by jurisdiction, while rates of daily smoking and TB infection showed negative correlation (**Supplementary Table 13**). Mortality risk factors for OCCs and OPCs in males are presented in **Table 2**.

**Table 2:** Poisson regression analysis of risk factors for lip, oral cavity, and pharyngeal cancers (C00-C14) age-standardized incidence and mortality for males in the Russian Federation, 2008-2018.

Incidence Rate Males C00-C14 (Univariate Model)					
Predictor	Coefficient ß	I.R.R.	CIs	S.E.	*p-*value
Rare alcohol consumption	-**0.01**	0.992	0.986-0.999	0.003	0.02
Weekly alcohol consumption (once per week)	0.02	1.018	1.007-1.029	0.006	<0.005
Weekly alcohol consumption (2-3 times per week)	0.06	1.062	1.039-1.085	0.01	<0.0001
Smoking (<10 cigarettes/day)	**-0.01**	0.988	0.980-0.996	0.004	0.004
TB infection	**-0.003**	0.997	0.995-0.999	0.001	<0.005
Syphilis infection	**-0.004**	0.996	0.993-0.999	0.002	0.007
Gonorrhea infection	**-0.005**	0.995	0.992-0.998	0.001	<0.005
Sale of alcohol content <9%	0.01	1.010	1.000-1.020	0.005	0.039
Incidence Rate Males C00-C14 (Multivariate Model)					
Weekly alcohol consumption (2-3 times per week)	0.07	1.069	1.037-1.102	0.02	<0.0001
Alcohol induced psychosis	0.001	1.002	1.000-1.003	0.0007	0.03
Gonorrhea infection	**-0.005**	0.995	0.992-0.998	0.002	0.002

Mortality Rate Males C00-C14 (Univariate Model)					
Predictor	Coefficient ß	I.R.R.	CIs	S.E.	*p-*value
Any alcohol consumption	0.01	1.013	1.009-1.018	0.002	<1E-8
Rare alcohol consumption	0.02	1.016	1.010-1.022	0.003	<1E-7
Monthly alcohol consumption	**-0.04**	0.961	0.952-0.971	0.005	<1E-13
Weekly alcohol consumption (once per week)	**-0.01**	0.986	0.976-0.996	0.005	<0.001
Never smoking	0.02	1.025	1.020-1.031	0.003	<1E-15
Ex-smoking	**-0.04**	0.965	0.950-0.980	0.008	<0.00001
Rare smoking	**-0.04**	0.965	0.943-0.986	0.01	0.002
Daily smoking	**-0.03**	0.968	0.961-0.974	0.003	<1E-15
Tuberculosis infection	**-0.007**	0.993	0.991-0.995	0.001	<1E-14
HIV infection	0.003	1.003	1.002-1.004	0.0004	<1E-11
Syphilis infection	**-0.005**	0.995	0.992-0.997	0.001	<0.001
Gonorrhea infection	**-0.01**	0.990	0.988-0.993	0.001	<1E-14
Sale of alcohol content <9%	0.02	1.022	1.010-1.034	0.006	<0.001
Mortality Rate Males C00-C14 (Multivariate Model)					
Weekly alcohol consumption (2-3 times per week)	0.06	1.060	1.029-1.090	0.01	<0.0001
Gonorrhea infection	**-0.004**	0.996	0.992-0.999	0.002	0.02

Bolded parameter estimates denote a negative association. Coefficient ß indicates regression coefficient. I.R.R. denotes the incidence rate ratios. S.E. denotes standard error. CIs indicate confidence intervals. p-values <0.05 were considered significant. Only significant values are shown.

Poisson univariate or multivariate regression models did not identify any significant risk factors for OCPCs (C00-C14) incidence in females (data not shown). Rate of daily smoking was shown to have a positive relationship on OCC incidence in the multivariate backwards stepwise regression model (**Table 3**). However, for OPC incidence (C10-C13), rate of ‘any’ alcohol consumption was the only risk factor identified as significant in the univariate model (**Table 3**). Mortality risk factors for OCPCs in females were negatively predicted by ‘never’ smoking, and positively predicted by daily smoking (**Table 3**). In multivariate analysis, never smoking was identified as a negative predictor.

**Table 3:** Poisson regression analysis of risk factors for lip and oral cavity cancers (C00-C09) age-standardized incidence and pharyngeal cancer (C10-C13) (separately) and combined (C00-14) age-standardized mortality for females in the Russian Federation, 2008-2018.

Incidence Rate Females C00-C09 (Multivariate Model)								
Predictor	Coefficient ß	I.R.R.		CIs			S.E.	*p-*value
Daily smoking	0.02	1.024		1.002-1.045			0.01	0.03
Incidence Rate Females C10-C13 (Univariate Model)								
Any alcohol consumption	**-0.02**	0.983		0.971-0.997			0.01	0.01

Mortality Rate Females C00-C14 (Univariate Model)								
Predictor	Coefficient ß	I.R.R.		CIs			S.E.	*p-*value
Never smoking	**-0.02**	0.982		0.966-0.999			0.009	0.04
Daily smoking	0.03	1.027		1.003-1.051			0.01	0.03
Mortality Rate Females C00-C14 (Multivariate Model)								
Never smoking	**-0.02**	0.980		0.962-0.999			0.01	0.04

Bolded parameter estimates denote a negative association. Coefficient ß indicates regression coefficient. I.R.R. denotes the incidence rate ratios. S.E. denotes standard error. CIs indicate confidence intervals. p-values <0.05 were considered significant. Only significant values are shown.

## Discussion

Russia is a large and diverse country with different culture, ethnicities, and SES by region, which allows for a close examination of associated risk factors and geographic distribution trends. Our results demonstrate that overall, Russia has a significant OCC and OPC burden with increasing OCC rates in females and OPC for both sexes. In fact, the Russian Federation appeared to rank highly in terms of ASIR for males (10^th^ out of 41 comparison countries available) whereas for females, it ranked 27^th^ (based on 2012 estimates). The ASMR of OCPC (4.44/100,000 deaths) ranked 2^nd^ with only UK, Scotland reporting a higher rate (2). In our study, men constituted 76% of all OCC and OPC cases between 2007-2018. Our findings are consistent with a study that demonstrated that while men comprised 74.9% of all OCCs and OPCs in Siberia and the Far East, the rate of increase in incidence was 3.4-fold higher in females (21). Of note, male ASMR from both OCCs and OPCs is consistently higher than in females, even up to 12-times in certain regions, such as the Bryansk Oblast. The reasons for this are equivocal and likely multifaceted, but may be related to men on average being less likely to seek medical care than women, and thus presenting at later stages of the disease (22). It is also unclear why there appears to be a predilection towards southwestern regions in ASMR for OCCs and OPCs for both sexes.

The geographic distribution of age-adjusted incidence of OCCs across Russia demonstrated a north-to-south gradient. Specifically, Bryansk Oblast, Chechen Republic, and Republic of Mordovia, all located in the western part of the country, had very high incidence rates (approximately 1.7-fold greater than the national average). Lip SCC accounts for approximately 25-30% of all OCCs (23). UVR is a strong risk factor in its pathogenesis, with increasing intensity towards the equator, potentially explaining our findings, such as in Chechen Republic (12.82; 95%CI 10.10-15.54), Altai Krai (9.76; 95% CI 8.53 - 10.99), Orenburg oblast (9.89 95% CI 8.51-11.28), Zabaykalsky Krai (9.34; 95% CI 7.35-11.34), Amur oblast (9.20; 95% CI 7.03-11.38), and Sakhalin oblast (9.52; 95% CI 6.72-12.32), which are the southern parts of the country. Notably, some jurisdictions within the Northern Caucasian Federal District, the southernmost district – Republics of Dagestan and Ingushetia – did not have particularly high incidences, despite neighboring Chechen Republic geographically. This was also a trend noted in our previous analyses (24, 25). Unfortunately, these regions have also experienced significant political unrest over the years, which could impact data collection and contribute to the low rates reported.

Geographic mapping of ASIR of OPCs did not demonstrate appreciable patterns. Nevertheless, certain jurisdictions were notable for incidence rates 1.9-2.5 times that of the national average: Chechen Republic had the highest incidence rate in the country, followed by Oryol, Sakhalin, and Kursk Oblasts. While Oryol and Kursk are in the Central Federal District and are relatively urbanized, Chechen Republic and Sakhalin Oblast are more rural, and its residents may thus have less access to medical care. According to the Organization of Economic Co-operation and Development, an intergovernmental organization for socioeconomic policy development, a rural community has a population density of <150 people/km^2^ (26). Approximately 62% of Chechen Republic population lived rurally in 2021 (27), with a population density of 87/km^2^. Similarly, Sakhalin Oblast is in the Far East Federal District, with a population of 6/km^2^. A Russian study previously demonstrated that Sakhalin Oblast had one of the highest crude incidences of OCCs and OPCs for both sexes across all of Siberia and the Far East (21). Although not comprehensively explored in our study, rural areas should be evaluated for incidence rates of OCCs and OPCs, as prior studies have found a lower rate of decline in incidence rates for HPV preventable cancers in rural regions in comparison to urban settings (28). Cancers associated with modifiable factors (i.e., tobacco and HPV) (29–31) had higher incidence in rural areas of the United States, as were cancers with either new or established screening modalities including lung, cervical, and colorectal cancers (28).

Russia is observing an overall increase in incidence of OPCs (with stable rates of OCCs) over the studied time period (2007-2018). During a similar time period (2007–2016), the total incidence of OPC in the United States has increased significantly (32). The trend was hypothesized to be due to increased sexual behavior (*i.e.*, oral-genital contact) and multiple partners (33). While the present study does not have data on HPV infection rates in the Russian population, we utilized STIs as potential surrogate markers of HPV infection (34, 35) in relation to OCC and OPC incidence and/or mortality. Interestingly, our study shows that STIs, such as gonorrhea, are less prevalent in new diagnoses of OCC and OPC in males, possibly suggesting that non-HPV carcinogenesis is a more dominant pathway in this demographic.

A large-scale multicenter study using the International Head and Neck Cancer Epidemiology Consortium data of Europe, North America, and Latin America has shown that a low frequency of smoking was strongly associated with an increased risk of developing head and neck cancers, even among never alcohol drinkers (36). Our study echoed these findings, such that daily smoking was a strong positive predictor of incidence of OCCs in Russian females, while never smoking was a negative predictor of both OCC and OPC mortality in females. While the incidence of OCCs in males is approximately 4-fold greater compared to females, the rate of cancer in females has been steadily increasing while decreasing for males. In 2001, a WHO-conducted survey revealed that Russia had one of the highest smoking prevalence rates in the world (37), and the second largest tobacco market by volume of sales in 2014, despite having only 2% of the world’s population (38). For women, between 1992-2003, age‐standardized prevalence of smoking more than doubled from 6.9% to 15% (p<0.001) (39). The trend was significant for all age groups, except for those ≥65 years. This could partially be exacerbated by tobacco marketing in Russia targeted specifically towards women: advertisements often depict themes of female independence, beauty, and citing the cigarettes as ‘slim’ and ‘light’, which has been shown to mislead the consumer about the consequences of smoking (40). This social tendency corresponds to the increased OCC and OPC rates in females observed in our study, given a 20-30 year latency period for carcinogenesis. Future studies in Russia should explore the effect of duration of smoking on OCC and OPC development, as duration may be a more significant factor than frequency (41). Smoking is a public health concern in Russia, and control strategies should continue to be optimized to reduce the rates of OCCs and OPCs.

A recent study presented strong evidence of an independent, direct causal effect of alcohol consumption on OCC and OPC carcinogenesis (42). A meta-analysis previously described a dose-dependent response in alcohol consumption in OCC and OPC development, noting an increased risk even with light drinking (RR 1.1; 95%CI 1.0-1.3 for ≤12.5 grams/day, RR 5.1; 95%CI 4.3-6.1 for >50 grams/day) (43). We identified frequent weekly alcohol consumption (2-3 times per week) as the most persistent significant predictor in incidence and mortality of OCC and OPC for both sexes in the Russian population. Though our study does not measure a dose-dependent response, the persistence of this variable across all measures, suggests that even light drinking may be a risk factor for carcinogenesis. Additionally, between 2004-2011, Russia saw an introduction of strict alcohol policies, resulting in significant decrease in alcohol use (44). This may help explain the decreasing trend in OCCs in males observed in our study. Finally, we were also able to demonstrate that regional sales of alcohol beverages with alcohol content <9% may be associated with higher rates of pharyngeal cancers in Russian males. Combined with our findings of ‘lighter’ frequency of alcohol consumption being a risk factor, this may suggest that ‘lighter’ may not necessarily be better, both from the perspective of frequency and alcohol content. Reduction of OCC and OPC merits a biopsychosocial approach in managing alcohol consumption, particularly in communities where crude rates of consumption and alcohol sales are high.

We performed a retrospective populational study utilizing open-source government data for the Russian Federation population between 2007-2018. There are several important limitations to our study. First, standards of data reporting and registry likely vary between regions, introducing possible reporting inaccuracies. Unfortunately, data was available until the year 2018 so more recent trends in incidence/mortality and geographic distribution could not be assessed. Moreover, data on smoking and alcohol consumption were acquired from self-reported surveys, potentially introducing recall bias. Finally, the risk factor data are populational, combining males and females, which may affect the sensitivity of identifying a risk factor in the stratified analysis of cancer rates in males and females. Given we identified significant differences in incidence and mortality between females and males, further studies exploring risk factors by sex would benefit in explaining such striking findings. The data found in this study is focused on elucidating trends in the country and may not be directly generalizable to the world population. However, similar risk factors have been shown to play a role in carcinogenesis in other parts of the world.

Russia has a significant and growing burden in OCCs and OPCs which warrants prompt introduction of robust, national HPV vaccination programs for both sexes, and continued comprehensive implementation of interventions to mitigate modifiable risk factors, such as alcohol consumption and smoking. This study encourages collaboration between Russia and the scientific community at-large to advance global health initiatives in reducing the worldwide burden of OCCs and OPCs. Russia has the economic potential to mobilize resources required to reduce the burden of preventable cancers. It is regrettable that due to the ongoing military conflict, significant resources may not be allocated to achieving these goals in serving the population. We call on the Russian scientific and medical community to mobilize and to focus on improving health.

## Data availability statement

The original contributions presented in the study are included in the article/**Supplementary Material**. Further inquiries can be directed to the corresponding author.

## Ethics statement

Ethical review and approval was not required for the study on human participants in accordance with the local legislation and institutional requirements. Written informed consent from the participants’ legal guardian/next of kin was not required to participate in this study in accordance with the national legislation and the institutional requirements.

## Author contributions

Conceptualization, AM, AZ, and IL. Methodology, AM, VN, EP, JL, ER, and AZ. Software, AZ, VN, and EN. Validation, AM, VN, EP, JL, and ER. Formal analysis, AM, EP, VN, and JL. Resources, AZ, EN, and IL. Data curation, AM, VN, and AZ. Writing—original draft preparation, AM and EP. Writing—review and editing, AM, VN, EP, JL, ER, AZ, EN, and IL. Visualization, AM and EP. Supervision, EN and IL. Project administration, IL. Funding acquisition, IL. All authors contributed to the article and approved the submitted version.
