# Supplementary material for: Burden and geographic distribution of oral cavity and oropharyngeal cancers in the Russian Federation
Source: Front Oncol. 2023 Aug 3;13:1197287. doi: 10.3389/fonc.2023.1197287 (PMC10435750; doi:10.3389/fonc.2023.1197287)

Supplementary Appendix

Supplementary Table 1. Risk factors studied for OCC and OPC pathogenesis, with associated data sources.

Risk factor category	Risk factor(s) studied	Data source
Alcohol	alcohol consumption ‘at all’/any, ‘rarely’, ‘monthly’ (once per month), ‘weekly, infrequent’ (once per week), ‘weekly, frequent’ (two to three times per week), ‘daily’	Federal State Statistics Service (Rosstat), 2016
	sales of alcohol per region, stratified by <9% or >9% alcohol content in beverages	the Center of Investigation of Federal and Regional Alcohol Market, 2019
	alcohol-induced psychosis (alone) and with syndrome of dependency	Ministry of Health of the Russian Federation Monitoring and Analysis Department, 2014-2015
Smoking	‘never smoked’, ‘ex-smoker’, ‘rarely smoke’, ‘smoke daily’, ‘smoke <five cigarettes/day’, ‘smoke <ten cigarettes/day’, ‘smoke one or fewer packs/day’, ‘smoke one or more packs/day’	Federal State Statistics Service (Rosstat), 2016
Infections	human immunodeficiency virus (HIV), syphilis, gonorrhea, tuberculosis (TB)	Ministry of Health of the Russian Federation Monitoring and Analysis Department, 2014-2015

Supplementary Table 2. Average annual cases and crude rates by age group for lip and oral cavity cancers (OCC) (C00-C09) in the Russian Federation, 2007-2018. Both the absolute number and percent per age group are presented.

Age	Males			Females			Both sexes		
	#	%	crude rate	#	%	crude rate	#	%	crude rate
0-19	13.3	0.2	0.08 (0.03 - 0.17)	11.2	0.4	0.07 (0.03 - 0.16)	24.5	0.2	0.08 (0.04 - 0.14)
20-29	33.5	0.4	0.29 (0.17 - 0.46)	31.7	1	0.28 (0.16 - 0.46)	65.2	0.6	0.29 (0.20 - 0.40)
30-39	196	2.5	1.81 (1.47 - 2.20)	104.1	3.3	0.94 (0.70 - 1.23)	300.1	2.7	1.37 (1.16 - 1.61)
40-49	836.8	10.6	8.73 (7.91 - 9.61)	261.6	8.4	2.51 (2.09 - 2.97)	1098.4	10.0	5.48 (5.03 - 5.97)
50-59	2516.4	31.9	26.49 (25.04 - 28.01)	639.2	20.5	5.47 (4.88 - 6.11)	3155.6	28.7	14.90 (14.16 - 15.66)
60-69	2494.3	31.7	45.63 (43.10 - 48.25)	735.7	23.6	8.90 (8.01 - 9.87)	3229.9	29.4	23.53 (22.38 - 24.71)
70-79	1367.3	17.4	45.87 (42.46 - 49.46)	783.8	25.2	12.41 (11.21 - 13.71)	2151.1	19.6	23.14 (21.77 - 24.58)
≥80	209.9	2.7	41.83 (34.18 - 50.62)	273.5	8.8	16.82 (14.11 - 19.88)	483.4	4.4	22.72 (19.93 - 25.78)
Total	7877.3	100	11.82 (11.45 - 12.20)	3114.2	100	4.03 (3.83 - 4.24)	10991.5	100	7.64 (7.43 - 7.84)

Supplementary Table 3. Average annual cases and crude rates by age group for oropharyngeal cancer (OPCs) (C10-13) in the Russian Federation, 2007-2018. Both the absolute number and percent per age group are presented.

Age	Males			Females			Both sexes		
	#	%	crude rate	#	%	crude rate	#	%	crude rate
0-19	17.3	0.4	0.11 (0.05 - 0.21)	11.8	1.7	0.08 (0.03 - 0.17)	29.1	0.6	0.09 (0.05 - 0.15)
20-29	23.6	0.6	0.21 (0.11 - 0.36)	14.1	2.0	0.13 (0.05 - 0.25)	37.7	0.8	0.17 (0.10 - 0.26)
30-39	82.4	2.0	0.76 (0.55 - 1.03)	35.6	5.1	0.32 (0.19 - 0.50)	118	2.4	0.54 (0.41 - 0.69)
40-49	447.8	10.7	4.67 (4.07 - 5.32)	95.8	13.8	0.92 (0.68 - 1.21)	543.5	11.1	2.71 (2.40 - 3.06)
50-59	1530.8	36.5	16.12 (14.98 - 17.31)	210.5	30.4	1.80 (1.47 - 2.18)	1741.3	35.6	8.22 (7.68 - 8.79)
60-69	1468.3	35	26.86 (24.93 - 28.89)	179.4	25.9	2.17 (1.74 - 2.67)	1647.8	33.7	12.00 (11.19 - 12.86)
70-79	542.5	12.9	18.20 (16.08 - 20.51)	97.8	14.1	1.55 (1.15 - 2.04)	640.3	13.1	6.89 (6.15 - 7.69)
≥80	43	1.0	8.56 (5.35 - 12.93)	23.5	3.4	1.45 (0.75 - 2.51)	66.5	1.4	3.13 (2.16 - 4.36)
Total	4198.5	100	6.30 (6.03 - 6.58)	692.1	100	0.90 (0.80 - 1.00)	4890.6	100	3.40 (3.26 - 3.54)

Supplementary Table 4. Average annual cases and crude rates by age group for lip, oral cavity and oropharyngeal cancers combined (OCPCs) (C00-14) in the Russian Federation, 2007-2018. Both the absolute number and percent per age group are presented.

Age	Males			Females			Both sexes		
	#	%	crude rate	#	%	crude rate	#	%	crude rate
0-19	30.6	0.3	0.19 (0.11 - 0.31)	23	0.6	0.15 (0.08 - 0.27)	53.6	0.3	0.17 (0.11 - 0.25)
20-29	57.1	0.5	0.50 (0.33 - 0.71)	45.8	1.2	0.41 (0.26 - 0.61)	102.8	0.6	0.45 (0.34 - 0.59)
30-39	278.4	2.3	2.57 (2.16 - 3.03)	139.7	3.7	1.26 (0.98 - 1.59)	418.1	2.6	1.91 (1.66 - 2.19)
40-49	1284.6	10.6	13.40 (12.37 - 14.48)	357.3	9.4	3.42 (2.94 - 3.96)	1641.9	10.3	8.20 (7.64 - 8.78)
50-59	4047.2	33.5	42.61 (40.76 - 44.53)	849.7	22.3	7.27 (6.59 - 8.00)	4896.8	30.8	23.12 (22.20 - 24.06)
60-69	3962.6	32.8	72.49 (69.30 - 75.78)	915.1	24	11.08 (10.08 - 12.14)	4877.7	30.7	35.53 (34.12 - 36.98)
70-79	1909.8	15.8	64.07 (60.03 - 68.29)	881.7	23.2	13.96 (12.68 - 15.34)	2791.4	17.6	30.03 (28.46 - 31.66)
≥80	252.8	2.1	50.40 (41.96 - 59.97)	297	7.8	18.27 (15.43 - 21.45)	549.9	3.5	25.84 (22.86 - 29.10)
Total	12075.8	100	18.12 (17.66 - 18.59)	3806.3	100	4.92 (4.70 - 5.15)	15882.1	100	11.03 (10.79 - 11.28)

Supplementary Table 5. Average annual cases and crude rates of mortality by age group for lip, oral cavity and oropharyngeal cancers combined (OCPCs) (C00-14) in the Russian Federation, 2007-2018. Both the absolute number and percent per age group are presented.

Age	Males			Females			Both sexes		
	#	%	crude rate	#	%	crude rate	#	%	crude rate
0-19	5.9	0.1	0.04 (0.01 - 0.10)	5.1	0.3	0.03 (0.01 - 0.10)	11	0.1	0.04 (0.01 - 0.08)
20-29	20.3	0.3	0.18 (0.09 - 0.32)	15.2	0.8	0.14 (0.06 - 0.27)	35.4	0.4	0.16 (0.09 - 0.25)
30-39	128.2	1.7	1.18 (0.91 - 1.51)	48.7	2.7	0.44 (0.28 - 0.65)	176.8	1.9	0.81 (0.65 - 0.99)
40-49	716.3	9.5	7.47 (6.71 - 8.29)	157.2	8.7	1.51 (1.19 - 1.88)	873.5	9.4	4.36 (3.96 - 4.79)
50-59	2572.3	34.2	27.08 (25.61 - 28.62)	386.1	21.4	3.30 (2.85 - 3.81)	2958.3	31.7	13.97 (13.26 - 14.70)
60-69	2600.3	34.5	47.57 (44.99 - 50.25)	424.1	23.5	5.13 (4.46 - 5.87)	3024.3	32.4	22.03 (20.92 - 23.18)
70-79	1197.3	15.9	40.17 (36.99 - 43.54)	413.5	22.9	6.55 (5.68 - 7.51)	1610.8	17.3	17.33 (16.14 - 18.58)
≥80	143.3	1.9	28.56 (22.32 - 35.94)	176.1	9.8	10.83 (8.68 - 13.33)	319.4	3.4	15.01 (12.76 - 17.53)
Total	7527.1	100	11.29 (10.93 - 11.66)	1802	100	2.33 (2.18 - 2.49)	9329.1	100	6.48 (6.29 - 6.67)

Supplementary Table 6. Crude and age-standardized incidence rates of lip and oral cavity cancers (OCC) (C00-C09) stratified by males, females, and both, in the Russian Federation, 2007-2018. Calculations performed based on data available by age group. Age-standardization performed according to the World Health Organization (WHO) Standard Population 2000-2025.

Year	C00-C09 crude incidence/100,000 (95%CIs)			C00-C09 age-standardized incidence/100,000 (95%CIs)		
	Male	Female	Both	Male	Female	Both
2007	11.42 (11.16-11.68)	3.74 (3.6-3.88)	7.29 (7.16-7.44)	9.78 (9.56-10.01)	2.29 (2.2-2.38)	5.23 (5.13-5.34)
2008	11.75 (11.49-12.01	3.62 (3.49-3.76)	7.38 (7.24-7.53)	9.97 (9.74-10.19)	2.2 (2.11-2.28)	5.26 (5.16-5.37)
2009	11.85 (11.59-12.12)	3.76 (3.62-3.9)	7.5 (7.36-7.65)	10.1 (9.87-10.33)	2.29 (2.2-2.38)	5.36 (5.25-5.46)
2010	11.76 (11.5-12.03)	3.92 (3.78-4.07)	7.55 (7.41-7.69)	9.83 (9.61-10.05)	2.38 (2.29-2.47)	5.33 (5.22-5.43)
2011	11.46 (11.2-11.72)	3.97 (3.83-4.12)	7.43 (7.29-7.58)	9.25 (9.03-9.46)	2.3 (2.21-2.38)	5.1 (5-5.2)
2012	11.44 (11.19-11.7)	3.8 (3.67-3.95)	7.34 (7.2-7.48)	9.22 (9.01-9.43)	2.23 (2.15-2.32)	5.04 (4.94-5.14)
2013	11.69 (11.43-11.95)	3.8 (3.66-3.94)	7.45 (7.31-7.59)	9.27 (9.06-9.47)	2.25 (2.16-2.33)	5.08 (4.98-5.18)
2014	12.05 (11.78-12.31)	3.99 (3.85-4.13)	7.72 (7.58-7.87)	9.45 (9.24-9.66)	2.31 (2.22-2.4)	5.2 (5.11-5.3)
2015	12.02 (11.76-12.29)	4.3 (4.15-4.45)	7.88 (7.73-8.02)	9.31 (9.1-9.51)	2.54 (2.45-2.63)	5.27 (5.17-5.37)
2016	12.12 (11.86-12.38)	4.41 (4.26-4.56)	7.98 (7.84-8.13)	9.28 (9.07-9.48)	2.58 (2.49-2.67)	5.3 (5.2-5.4)
2017	11.97 (11.71-12.23)	4.37 (4.23-4.52)	7.89 (7.75-8.04)	9.11 (8.91-9.31)	2.54 (2.45-2.63)	5.21 (5.11-5.3)
2018	12.27 (12.01-12.54)	4.61 (4.46-4.76)	8.16 (8.02-8.31)	9.24 (9.04-9.44)	2.68 (2.59-2.77)	5.36 (5.26-5.45)
Average 2007-2018	11.82 (11.64-12.00)	4.02 (3.82-4.23)	7.63 (7.45-7.81)	9.48 (9.27-9.70)	2.38 (2.28-2.48)	5.23 (5.16-5.30)

Supplementary Table 7. Crude and age-standardized incidence rates of oropharyngeal cancers (OPC) (C10-C13) stratified by males, females, and both, in the Russian Federation, 2007-2018. Calculations performed based on data available by age group. Age-standardization performed according to the World Health Organization (WHO) Standard Population 2000-2025.

Year	C10-C13 crude incidence/100,000 (95%CIs)			C10-C13 age-standardized incidence/100,000 (95%CIs)		
	Male	Female	Both	Male	Female	Both
2007	5.67 (5.49-5.85	0.73 (0.68-0.8)	3.02 (2.93-3.11)	4.76 (4.61-4.92)	0.52 (0.47-0.56)	2.25 (2.18-2.31)
2008	5.65 (5.47-5.83)	0.77 (0.71-0.84)	3.03 (2.94-3.12)	4.75 (4.6-4.91)	0.58 (0.53-0.63)	2.28 (2.21-2.35)
2009	5.99 (5.806.18)	0.84 (0.78-0.91)	3.22 (3.13-3.32)	5.03 (4.87-5.19)	0.63 (0.58-0.68)	2.4 (2.33-2.47)
2010	5.98 (5.79-6.17)	0.84 (0.78-0.91)	3.22 (3.13-3.31)	4.94 (4.79-5.1)	0.59 (0.54-0.64)	2.36 (2.29-2.43)
2011	5.72 (5.54-5.91)	0.79 (0.73-0.86)	3.07 (2.98-3.16)	4.57 (4.42-4.71)	0.54 (0.5-0.59)	2.19 (2.13-2.26)
2012	6.05 (5.86-6.24)	0.84 (0.78-0.91)	3.25 (3.16-3.34)	4.81 (4.66-4.96)	0.57 (0.53-0.62)	2.31 (2.24-2.38)
2013	6.11 (5.93-6.31)	0.85 (0.79-0.92)	3.29 (3.2-3.38)	4.78 (4.64-4.93)	0.58 (0.53-0.62)	2.31 (2.25-2.38)
2014	6.49 (6.3-6.69)	0.92 (0.86-0.99)	3.5 (3.41-3.6)	4.99 (4.84-5.14)	0.62 (0.58-0.67)	2.43 (2.36-2.5)
2015	6.87 (6.68-7.07)	0.95 (0.89-1.03)	3.7 (3.6-3.8)	5.22 (5.07-5.37)	0.64 (0.59-0.68)	2.52 (2.46-2.59)
2016	6.84 (6.65-7.04)	1 (0.93-1.07)	3.71 (3.61-3.81)	5.14 (4.99-5.29)	0.65 (0.6-0.7)	2.5 (2.44-2.57)
2017	6.86 (6.66-7.06)	1.0 (1.01-1.16)	3.76 (3.66-3.86)	5.11 (4.97-5.26)	0.72 (0.67-0.77)	2.53 (2.46-2.6)
2018	7.28 (7.07-7.48)	1.1 (1.02-1.17)	3.96 (3.86-4.06)	5.41 (5.25-5.56)	0.72 (0.67-0.77)	2.66 (2.59-2.73)
Average 2007-2018	6.29 (5.94-6.64)	0.89 (0.82-0.97)	3.39 (3.19-3.60)	4.96 (4.81-5.11)	0.61 (0.57-0.65)	2.40 (2.31-2.48)

Supplementary Table 8. Crude and age-standardized incidence rates of lip, oral and oropharyngeal cancers (OCCs and OPCs) (C00-C14) stratified by males, females, and both, in the Russian Federation, 2007-2018. Calculations performed based on data available by age group. Age-standardization performed according to the World Health Organization (WHO) Standard Population 2000-2025.

Year	C00-C14 crude incidence/100,000 (95%CIs)			C00-C14 age-standardized incidence/100,000 (95%CIs)		
	Male	Female	Both	Male	Female	Both
2007	17.09 (16.77-17.41)	4.48 (4.33 - 4.63)	10.31 (10.15 - 10.48)	14.55 (14.27 - 14.82)	2.81 (2.71 - 2.91)	7.48 (7.36 - 7.60)
2008	17.40 (17.08 - 17.72)	4.39 (4.25 - 4.55)	10.41 (10.24 - 10.58)	14.72 (14.45 - 15.00)	2.78 (2.68 - 2.88)	7.54 (7.42 - 7.67)
2009	17.84 (17.52 - 18.17)	4.60 (4.45 - 4.75)	10.72 (10.55 - 10.90)	15.13 (14.85 - 15.41)	2.92 (2.81 - 3.02)	7.76 (7.63 - 7.89)
2010	17.74 (17.42 - 18.07)	4.77 (4.61 - 4.93)	10.77 (10.60 - 10.94)	14.77 (14.50 - 15.05)	2.97 (2.87 - 3.07)	7.69 (7.56 - 7.81)
2011	17.18 (16.86 - 17.50)	4.76 (4.61 - 4.92)	10.50 (10.34 - 10.67)	13.81 (13.55 - 14.07)	2.84 (2.74 - 2.93)	7.29 (7.17 - 7.41)
2012	17.49 (17.18 - 17.81)	4.64 (4.49 - 4.80)	10.59 (10.42 - 10.76)	14.03 (13.77 - 14.29)	2.81 (2.71 - 2.90)	7.35 (7.23 - 7.47)
2013	17.80 (17.48 - 18.13)	4.65 (4.50 - 4.81)	10.74 (10.57 - 10.91)	14.05 (13.80 - 14.31)	2.82 (2.72 - 2.92)	7.40 (7.28 - 7.52)
2014	18.54 (18.21 - 18.87)	4.91 (4.76 - 5.07)	11.22 (11.05 - 11.40)	14.44 (14.18 - 14.70)	2.93 (2.84 - 3.03)	7.63 (7.51 - 7.75)
2015	18.90 (18.57 - 19.23)	5.25 (5.09 - 5.42)	11.57 (11.40 - 11.75)	14.53 (14.27 - 14.78)	3.18 (3.08 - 3.28)	7.80 (7.68 - 7.92)
2016	18.96 (18.64 - 19.29)	5.41 (5.25 - 5.57)	11.69 (11.51 - 11.86)	14.42 (14.17 - 14.67)	3.23 (3.12 - 3.33)	7.81 (7.69 - 7.92)
2017	18.83 (18.50 - 19.16)	5.45 (5.29 - 5.62)	11.65 (11.48 - 11.83)	14.22 (13.97 - 14.47)	3.26 (3.16 - 3.36)	7.74 (7.62 - 7.86)
2018	19.55 (19.22 - 19.88)	5.70 (5.54 - 5.87)	12.12 (11.95 - 12.30)	14.64 (14.39 - 14.89)	3.40 (3.29 - 3.50)	8.01 (7.89 - 8.13)
Average 2007-2018	18.11 (17.59-18.62)	4.92 (4.65-5.19)	11.03 (10.64-11.41)	14.44 (14.21-14.68)	2.99 (2.86-3.13)	7.62 (6.49-7.76)

Supplementary Table 9. Crude and age-standardized mortality rates lip, oral cavity and oropharyngeal cancers (C00-C14) stratified by males, females, and both, in the Russian Federation, 2007-2018. Calculations performed based on data available by age group. Age-standardization performed according to the World Health Organization (WHO) Standard Population 2000-2025.

Year	C00-C14 crude mortality/100,000 (95%CIs)			C00-C14 age-standardized mortality/100,000 (95%CIs)		
	Male	Female	Both	Male	Female	Both
2007	10.15 (9.91-10.4)	2.05 (1.95-2.16)	5.8 (5.68-5.93)	8.65 (8.44-8.86)	1.24 (1.18-1.31)	4.19 (4.1-4.28)
2008	10.81 (10.56-11.07)	2.14 (2.04-2.25)	6.15 (6.03-6.28)	9.11 (8.89-9.32)	1.31 (1.24-1.37)	4.42 (4.32-4.51)
2009	10.91 (10.66-11.16)	2.12 (2.02-2.23)	6.19 (6.06-6.32)	9.21 (9-9.43)	1.27 (1.21-1.34)	4.43 (4.34-4.52)
2010	11.14 (10.88-11.39)	2.24 (2.14-2.35)	6.36 (6.22-6.49)	9.26 (9.04-9.48)	1.34 (1.28-1.41)	4.5 (4.4-4.59)
2011	11.24 (10.98-11.49)	2.27 (2.17-2.38)	6.42 (6.29-6.55)	9.04 (8.83-9.24)	1.31 (1.25-1.38)	4.45 (4.36-4.54)
2012	11.28 (11.03-11.54)	2.3 (2.19-2.41)	6.45 (6.32-6.59)	9.01 (8.81-9.22)	1.35 (1.29-1.42)	4.45 (4.35-4.54)
2013	11.49 (11.23-11.75)	2.37 (2.26-2.48)	6.59 (6.46-6.72)	9.03 (8.82-9.23)	1.38 (1.31-1.45)	4.5 (4.41-4.59)
2014	11.49 (11.23-11.75)	2.34 (2.24-2.46)	6.58 (6.45-6.71)	8.91 (8.71-9.11)	1.36 (1.3-1.43)	4.44 (4.35-4.53)
2015	11.8 (11.54-12.06)	2.37 (2.26-2.48)	6.73 (6.6-6.87)	9.02 (8.82-9.22)	1.38 (1.31-1.45)	4.49 (4.4-4.58)
2016	11.99 (11.73-12.25)	2.64 (2.53-2.75)	6.97 (6.84-7.11)	9.04 (8.84-9.24)	1.55 (1.48-1.62)	4.6 (4.51-4.69)
2017	11.46 (11.2-11.71)	2.49 (2.38-2.6)	6.65 (6.52-6.78)	8.57 (8.38-8.76)	1.42 (1.36-1.49)	4.35 (4.27-4.44)
2018	11.72 (11.46-11.98)	2.61 (2.5-2.73)	6.84 (6.7-6.97)	8.71 (8.51-8.9)	1.53 (1.46-1.6)	4.47 (4.38-4.56)
Av erage 2007-2018	11.29 (10.97-11.61)	2.33 (2.21-2.45)	6.48 (6.27-6.68)	8.96 (8.82-9.10)	1.37 (1.31-1.43)	4.44 (4.38-4.50)

Supplementary Table 10. Incidence of lip and oropharyngeal cancer (C00-C14) for 2012 (mid point of the study) comparing Russian Federation (based on our data) to other countries for which 2012 data was available from the International Agency for Research of Cancer (IARC). Russian Federation ranks 10th of 41 countries/regions listed. On the other hand, for females it ranks 27th of 41.

	**Males**		**Females**	
Country	**ASIR**	**Crude**	**ASIR**	**Crude**
**Russian Federation (our study data)**	**14.44**	**18.11**	**2.99**	**4.92**
Bulgaria	10.51	17.83	2.85	5.79
Belarus	20.03	27.85	2.23	4.32
Canada	10.15	16.44	3.83	6.94
Chile	1.28	1.58	0.40	0.52
China	12.20	21.13	4.60	8.22
Colombia	4.81	4.83	2.92	3.59

Croatia	15.28	25.84	2.79	5.79
Czechia	13.15	21.59	4.28	8.30
Denmark	13.90	23.71	6.02	10.83
Ecuador	2.14	2.10	1.61	1.62
Estonia	11.33	17.82	2.65	5.53
France	19.16	30.64	6.00	10.35
Germany	16.34	28.35	6.17	11.80
Iceland	4.70	6.84	5.39	8.77
India	24.43	25.64	9.20	9.67
Australia	15.11	22.43	5.73	9.45
Ireland	9.53	12.56	3.71	5.27
Israel	4.49	5.23	2.92	3.88
Italy	7.55	14.28	2.56	5.51
Republic of Korea	6.06	8.20	2.09	3.09
Kuwait: Kuwaiti	6.85	4.26	3.75	2.30
Latvia	14.06	22.13	2.08	4.53
Lithuania	13.03	19.47	2.29	4.78
Malta	7.38	11.97	5.21	9.49
The Netherlands	9.17	16.07	5.12	9.65
New Zealand	8.39	12.48	3.89	6.39
Norway	8.79	14.34	3.80	7.32
Poland	10.21	16.69	2.53	4.60
Slovenia	15.72	26.24	3.46	6.26
Sweden	7.45	13.61	4.51	8.73
Switzerland	12.90	21.89	5.59	10.62
Brazil	12.74	11.95	4.53	5.01
Thailand	9.69	13.14	3.68	5.30
Turkey	5.97	6.84	2.67	3.22

Uganda	12.98	4.27	5.47	2.04
Ukraine	16.77	24.06	2.14	4.31
UK, England and Wales	10.52	16.67	4.79	8.42
UK, Scotland	14.47	24.60	6.55	11.99
UK, Northern Ireland	11.56	17.44	5.32	8.50
USA	10.86	15.64	4.16	6.88

Data Source:

https://gco.iarc.fr/overtime/en/dataviz/trends?populations=84000&sexes=1_2&types=0&multiple_populations=0&mode=cancer&multiple_cancers=1&cancers=1&years=2009_2015

Supplementary Table 11. Age-standardized incidence rates (ASIR) (both sexes) of lip and oral cavity cancer (OCC) (C00-C09), oropharyngeal cancers (OPCs) (C10-C13), and lip, oral cavity, and oropharyngeal cancer combined (OCPCs) (C00-14) presented by jurisdiction of the Russian Federation as well as age-standardized mortality rates (ASMR), 2008-2018. Russian Federation total average population calculated based on the sum of all geographic areas (excluding Federal Districts).

Region	Average Total Population (2008-2018)	ASIR/100,000						ASMR/100,000	
		C00-09		C10-13		C00-14		C0014	
		Cases	Mean (95% CIs)	Cases	Mean (95% CIs)	Cases	Mean (95% CIs)	Cases	Mean (95% CIs)
Russian Federation	144544419	11048	7.64 (7.50 - 7.79)	4945	3.42 (3.33 - 3.52)	15993	11.06 (10.89 - 11.24)	9428	6.52 (6.39 - 6.65)
Central Federal District*	38343876	3004	7.33 (7.07 - 7.59)	1691	4.19 (3.99 - 4.39)	4694	11.51 (11.18 - 11.84)	3032	7.44 (7.17 - 7.70)
Belgorod Oblast	1541557	127	7.70 (6.36 - 9.04)	95	5.80 (4.63 - 6.96)	222	13.49 (11.71 - 15.26)	128	7.77 (6.43 - 9.12)
Bryansk Oblast	1247372	175	13.07 (11.13 - 15.00)	75	5.74 (4.44 - 7.03)	250	18.81 (16.48 - 21.14)	171	12.84 (10.92 - 14.77)
Vladimir Oblast	1410641	93	5.94 (4.74 - 7.15)	80	5.16 (4.03 - 6.29)	173	11.08 (9.43 - 12.73)	124	7.92 (6.53 - 9.32)
Voronezh Oblast	2319955	184	7.12 (6.09 - 8.15)	118	4.66 (3.82 - 5.51)	302	11.75 (10.43 - 13.08)	191	7.42 (6.37 - 8.47)
Ivanovo Oblast	1041461	103	8.91 (7.19 - 10.64)	49	4.32 (3.11 - 5.53)	151	13.23 (11.12 - 15.33)	84	7.32 (5.75 - 8.89)
Tver Oblast	1323145	136	9.10 (7.57 - 10.64)	53	3.66 (2.68 - 4.64)	189	12.77 (10.95 - 14.59)	117	7.89 (6.46 - 9.32)
Kaluga Oblast	1008182	98	8.87 (7.11 - 10.62)	68	6.21 (4.73 - 7.69)	165	15.06 (12.77 - 17.36)	104	9.47 (7.66 - 11.29)
Kostroma Oblast	659980	63	8.89 (6.71 - 11.08)	26	3.66 (2.25 - 5.07)	89	12.55 (9.95 - 15.15)	53	7.38 (5.38 - 9.37)
Kursk Oblast	1124783	139	11.09 (9.24 - 12.93)	78	6.33 (4.93 - 7.74)	217	17.41 (15.09 - 19.73)	125	9.99 (8.23 - 11.74)
Lipetsk Oblast	1158831	125	9.78 (8.06 - 11.50)	55	4.38 (3.22 - 5.53)	180	14.16 (12.09 - 16.23)	99	7.80 (6.26 - 9.33)
Moscow (city)	11845863	582	4.60 (4.23 - 4.97)	387	3.11 (2.80 - 3.42)	969	7.70 (7.21 - 8.18)	675	5.36 (4.96 - 5.76)

Moscow Oblast (does not include Moscow city)	7183226	466	6.34 (5.77 - 6.92)	224	3.06 (2.66 - 3.46)	690	9.40 (8.70 - 10.11)	496	6.77 (6.18 - 7.37)
Oryol Oblast	773627	101	11.74 (9.45 - 14.03)	67	7.94 (6.05 - 9.84)	169	19.66 (16.69 - 22.62)	95	11.05 (8.83 - 13.27)
Ryazan Oblast	1138632	128	9.76 (8.07 - 11.45)	70	5.52 (4.23 - 6.81)	198	15.25 (13.13 - 17.38)	104	7.96 (6.43 - 9.49)
Smolensk Oblast	965233	109	10.37 (8.43 - 12.31)	29	2.81 (1.79 - 3.82)	139	13.19 (10.99 - 15.38)	101	9.60 (7.73 - 11.46)
Tambov Oblast	1064270	117	9.53 (7.80 - 11.26)	49	4.10 (2.95 - 5.24)	166	13.63 (11.56 - 15.70)	94	7.69 (6.14 - 9.25)
Tula Oblast	1521458	151	8.51 (7.15 - 9.87)	84	4.90 (3.86 - 5.95)	235	13.39 (11.68 - 15.10)	155	8.79 (7.40 - 10.17)
Yaroslavl Oblast	1276337	107	7.61 (6.16 - 9.05)	82	5.98 (4.69 - 7.26)	189	13.55 (11.62 - 15.48)	116	8.31 (6.80 - 9.82)
Northwestern Federal District*	13746135	1067	7.46 (7.02 - 7.91)	465	3.26 (2.96 - 3.55)	1532	10.72 (10.18 - 11.26)	974	6.81 (6.39 - 7.24)
Arkhangelsk Oblast	1197638	101	8.38 (6.75 - 10.01)	46	3.74 (2.66 - 4.82)	147	12.12 (10.16 - 14.07)	83	6.84 (5.37 - 8.31)
Vologda Oblast	1193451	87	7.08 (5.59 - 8.57)	35	2.83 (1.89 - 3.78)	121	9.91 (8.15 - 11.68)	72	5.85 (4.50 - 7.21)
Kaliningrad Oblast	964570	71	7.33 (5.62 - 9.03)	23	2.41 (1.43 - 3.38)	94	9.73 (7.76 - 11.69)	60	6.21 (4.64 - 7.78)
Saint Petersburg	5054988	373	6.91 (6.21 - 7.61)	166	3.14 (2.66 - 3.62)	539	10.04 (9.20 - 10.89)	367	6.84 (6.14 - 7.54)
Leningrad Oblast	1748643	119	6.30 (5.17 - 7.43)	70	3.69 (2.82 - 4.56)	189	9.99 (8.56 - 11.41)	133	7.00 (5.81 - 8.19)
Murmansk Oblast	781953	68	9.62 (7.33 - 11.90)	24	3.17 (1.89 - 4.45)	92	12.75 (10.14 - 15.36)	53	7.37 (5.38 - 9.35)
Novgorod Oblast	622948	59	8.50 (6.33 - 10.66)	26	3.74 (2.29 - 5.19)	85	12.24 (9.63 - 14.84)	44	6.34 (4.47 - 8.22)
Pskov Oblast	659015	70	9.40 (7.20 - 11.61)	13	1.81 (0.83 - 2.78)	83	11.24 (8.83 - 13.66)	58	7.85 (5.83 - 9.87)
Republic of Karelia	641460	55	8.37 (6.17 - 10.57)	29	4.27 (2.70 - 5.83)	84	12.64 (9.93 - 15.34)	47	7.05 (5.03 - 9.06)
Komi Republic	881468	64	7.98 (6.02 - 9.94)	34	4.10 (2.72 - 5.47)	98	12.09 (9.69 - 14.48)	58	7.13 (5.29 - 8.97)
Southern Federal District*	15418086	1168	7.44 (7.02 - 7.87)	557	3.59 (3.29 - 3.88)	1725	11.03 (10.51 - 11.55)	967	6.18 (5.79 - 6.57)
Krasnodar Krai	5394290	426	7.68 (6.95 - 8.41)	232	4.21 (3.67 - 4.76)	658	11.89 (10.98 - 12.79)	362	6.53 (5.86 - 7.20)
Astrakhan Oblast	1014374	71	7.39 (5.67 - 9.10)	56	5.77 (4.25 - 7.28)	127	13.16 (10.87 - 15.45)	73	7.56 (5.83 - 9.30)
Volgograd Oblast	2564830	192	7.10 (6.10 - 8.11)	85	3.21 (2.53 - 3.89)	277	10.31 (9.10 - 11.52)	143	5.31 (4.44 - 6.18)
Rostov Oblast	4240121	308	6.90 (6.13 - 7.67)	107	2.41 (1.96 - 2.87)	415	9.32 (8.42 - 10.22)	258	5.79 (5.08 - 6.50)
Republic of Adygea	447391	30	6.75 (4.35 - 9.14)	15	3.30 (1.62 - 4.99)	45	10.04 (7.12 - 12.97)	34	7.66 (5.10 - 10.22)
Republic of Kalmykia	281330	15	6.20 (3.08 - 9.31)	6	2.35 (0.47 - 4.24)	21	8.54 (4.90 - 12.17)	11	4.55 (1.90 - 7.21)
North Caucasian Federal District*	9615035	521	7.15 (6.54 - 7.77)	268	3.63 (3.19 - 4.06)	789	10.78 (10.03 - 11.54)	387	5.34 (4.80 - 5.87)
Stavropol Krai	2779846	197	7.36 (6.33 - 8.38)	82	3.08 (2.41 - 3.75)	279	10.44 (9.22 - 11.66)	164	6.15 (5.21 - 7.10)
Republic of Ingushetia	469604	12	4.81 (2.14 - 7.49)	8	3.00 (0.94 - 5.05)	21	7.84 (4.46 - 11.23)	5	2.04 (0.30 - 3.79)
Republic of Dagestan	2945234	97	5.31 (4.26 - 6.37)	61	3.23 (2.42 - 4.04)	158	8.57 (7.23 - 9.90)	75	4.12 (3.18 - 5.05)
Kabardino-Balkaria Republic	867456	50	6.90 (4.99 - 8.82)	16	2.23 (1.15 - 3.31)	66	9.12 (6.92 - 11.33)	37	5.16 (3.50 - 6.82)
Republic of North Ossetia	704370	54	8.36 (6.14 - 10.58)	32	5.02 (3.28 - 6.76)	86	13.36 (10.54 - 16.18)	53	8.22 (6.01 - 10.44)
Karachay-Cherkessia Republic	462144	33	8.04 (5.29 - 10.78)	7	1.64 (0.40 - 2.88)	40	9.68 (6.66 - 12.69)	17	4.26 (2.26 - 6.26)

Chechen Republic	1347460	85	12.82 (10.10 - 15.54)	73	10.15 (7.82 - 12.49)	158	23.19 (19.57 - 26.80)	32	4.77 (3.11 - 6.43)
Volga Federal District*	29768118	2479	8.16 (7.84 - 8.48)	943	3.11 (2.91 - 3.30)	3421	11.27 (10.89 - 11.65)	1866	6.14 (5.86 - 6.42)
Nizhny Novgorod Oblast	3279157	302	8.53 (7.57 - 9.50)	146	4.18 (3.51 - 4.86)	448	12.71 (11.54 - 13.89)	241	6.82 (5.96 - 7.68)
Kirov Oblast	1321839	125	8.60 (7.09 - 10.10)	45	3.09 (2.18 - 3.99)	169	11.69 (9.93 - 13.44)	96	6.63 (5.31 - 7.95)
Samara Oblast	3199400	247	7.36 (6.45 - 8.28)	111	3.32 (2.70 - 3.94)	358	10.68 (9.58 - 11.79)	204	6.07 (5.23 - 6.90)
Orenburg Oblast	2021021	196	9.89 (8.51 - 11.28)	45	2.24 (1.58 - 2.89)	241	12.12 (10.59 - 13.65)	113	5.68 (4.63 - 6.72)
Penza Oblast	1358203	152	9.93 (8.35 - 11.51)	24	1.56 (0.93 - 2.19)	175	11.51 (9.81 - 13.22)	92	6.02 (4.79 - 7.26)
Permsky Krai	2643964	166	6.45 (5.47 - 7.43)	76	2.91 (2.26 - 3.57)	242	9.36 (8.18 - 10.54)	173	6.71 (5.71 - 7.71)
Saratov Oblast	2502779	222	8.30 (7.21 - 9.40)	83	3.15 (2.48 - 3.83)	305	11.46 (10.17 - 12.75)	141	5.30 (4.43 - 6.17)
Ulyanovsk Oblast	1270779	131	9.40 (7.79 - 11.02)	35	2.52 (1.69 - 3.36)	166	11.93 (10.11 - 13.74)	98	6.99 (5.60 - 8.37)
Republic of Bashkortostan	4064931	258	6.67 (5.85 - 7.48)	142	3.67 (3.07 - 4.28)	401	10.34 (9.33 - 11.35)	199	5.14 (4.43 - 5.86)
Mari El Republic	689720	57	8.37 (6.19 - 10.54)	13	1.92 (0.89 - 2.95)	70	10.26 (7.86 - 12.66)	39	5.65 (3.87 - 7.44)
Republic of Mordovia	815994	111	12.60 (10.26 - 14.95)	34	3.84 (2.54 - 5.13)	145	16.44 (13.76 - 19.12)	71	8.06 (6.18 - 9.93)
Republic of Tatarstan	3836269	304	8.08 (7.17 - 8.98)	101	2.69 (2.16 - 3.21)	404	10.77 (9.72 - 11.81)	241	6.42 (5.61 - 7.23)
Udmurt Republic	1518205	113	7.74 (6.32 - 9.17)	47	3.12 (2.22 - 4.01)	160	10.86 (9.17 - 12.54)	85	5.79 (4.57 - 7.02)
Chuvash Republic	1245859	96	7.81 (6.25 - 9.38)	41	3.38 (2.35 - 4.40)	137	11.19 (9.32 - 13.06)	74	6.01 (4.64 - 7.38)
Ural Federal District*	12256668	858	7.42 (6.93 - 7.92)	287	2.44 (2.16 - 2.72)	1145	9.85 (9.28 - 10.42)	630	5.43 (5.01 - 5.85)
Kurgan Oblast	885043	90	9.33 (7.40 - 11.26)	15	1.59 (0.79 - 2.39)	105	10.93 (8.84 - 13.02)	54	5.58 (4.09 - 7.07)
Sverdlovsk Oblast	4332369	318	7.31 (6.51 - 8.12)	142	3.26 (2.72 - 3.79)	460	10.57 (9.60 - 11.53)	257	5.90 (5.18 - 6.62)
Tyumen Oblast	3546718	194	7.08 (6.09 - 8.08)	59	2.00 (1.49 - 2.51)	253	9.02 (7.91 - 10.14)	134	4.80 (3.98 - 5.61)
Chelyabinsk Oblast	3492538	255	7.31 (6.41 - 8.20)	71	2.02 (1.55 - 2.50)	326	9.33 (8.32 - 10.35)	186	5.31 (4.55 - 6.07)
Siberian Federal District*	19146483	1474	8.20 (7.78 - 8.62)	586	3.21 (2.95 - 3.47)	2060	11.41 (10.91 - 11.90)	1182	6.54 (6.17 - 6.92)
Altai Krai	2401260	242	9.76 (8.53 - 10.99)	47	1.90 (1.36 - 2.44)	289	11.65 (10.31 - 12.99)	137	5.49 (4.57 - 6.41)
Krasnoyarsk Krai	2863574	219	8.30 (7.20 - 9.39)	131	4.84 (4.01 - 5.67)	350	13.15 (11.77 - 14.53)	202	7.60 (6.55 - 8.65)
Irkutsk Oblast	2430778	179	8.15 (6.96 - 9.35)	118	5.27 (4.32 - 6.22)	297	13.45 (11.92 - 14.98)	157	7.13 (6.02 - 8.25)
Kemerovo Oblast	2741220	208	7.68 (6.63 - 8.72)	79	2.87 (2.23 - 3.50)	286	10.54 (9.32 - 11.76)	192	7.05 (6.05 - 8.04)
Novosibirsk Oblast	2723089	219	8.11 (7.03 - 9.18)	94	3.48 (2.77 - 4.18)	313	11.58 (10.30 - 12.86)	181	6.71 (5.73 - 7.69)
Omsk Oblast	1977979	139	7.18 (5.99 - 8.38)	42	2.18 (1.52 - 2.84)	181	9.36 (7.99 - 10.72)	112	5.76 (4.69 - 6.83)
Tomsk Oblast	1064321	76	7.90 (6.13 - 9.68)	29	2.96 (1.89 - 4.04)	105	10.86 (8.79 - 12.93)	65	6.68 (5.05 - 8.31)
Zabaykalsky Krai (former Transbaykal region)**	1092143	84	9.34 (7.35 - 11.34)	14	1.53 (0.74 - 2.33)	98	10.82 (8.68 - 12.95)	56	6.21 (4.59 - 7.83)
Republic of Buryatia	975091	63	7.89 (5.94 - 9.84)	19	2.34 (1.30 - 3.39)	82	10.21 (8.00 - 12.42)	49	6.11 (4.40 - 7.82)
Altai Republic	212640	12	7.11 (3.01 - 11.21)	3	1.61 (0.00 - 3.51)	14	8.67 (4.17 - 13.16)	7	4.11 (1.00 - 7.21)
Tyva Republic	314964	10	5.91 (2.29 - 9.52)	2	1.02 (0.00 - 2.46)	12	6.84 (3.00 - 10.68)	8	4.42 (1.30 - 7.54)
Republic of Khakassia	535701	38	7.69 (5.24 - 10.14)	13	2.55 (1.16 - 3.94)	51	10.22 (7.41 - 13.04)	29	5.74 (3.64 - 7.85)

Far Eastern Federal District*	6443595	488	8.39 (7.64 - 9.13)	161	2.66 (2.25 - 3.07)	650	11.01 (10.17 - 11.86)	405	6.87 (6.20 - 7.54)
Primorsky Krai	1942175	160	8.42 (7.12 - 9.73)	36	1.86 (1.26 - 2.47)	196	10.25 (8.82 - 11.69)	130	6.80 (5.63 - 7.97)
Khabarovsk Krai	1347825	104	8.33 (6.73 - 9.94)	39	3.06 (2.10 - 4.02)	143	11.38 (9.52 - 13.25)	83	6.62 (5.20 - 8.05)
Amur Oblast	819448	69	9.20 (7.03 - 11.38)	12	1.49 (0.63 - 2.35)	80	10.63 (8.30 - 12.95)	47	6.16 (4.39 - 7.93)
Kamchatka Krai	322436	25	9.04 (5.53 - 12.56)	12	3.92 (1.69 - 6.15)	37	12.96 (8.80 - 17.12)	21	7.34 (4.21 - 10.48)
Magadan Oblast	151243	14	10.48 (5.05 - 15.92)	4	2.99 (0.21 - 5.76)	19	13.36 (7.31 - 19.42)	10	7.38 (2.89 - 11.88)
Sakhalin Oblast	495082	44	9.52 (6.72 - 12.32)	32	6.39 (4.16 - 8.62)	76	15.99 (12.40 - 19.59)	52	10.89 (7.93 - 13.86)
Chukotka Autonomous Okrug	50044	3	7.05 (0.00 - 15.72)	2	3.74 (0.00 - 9.65)	4	10.85 (0.34 - 21.37)	2	5.34 (0.00 - 12.75)
Sakha Republic (Yakutia)	957690	41	5.92 (4.12 - 7.73)	17	2.32 (1.23 - 3.40)	59	8.22 (6.12 - 10.32)	37	5.17 (3.49 - 6.84)
Jewish Autonomous Oblast	171373	15	9.54 (4.65 - 14.43)	2	1.32 (0.00 - 3.10)	17	10.78 (5.62 - 15.95)	12	7.50 (3.19 - 11.81)

***-**Data for Federal Districts averaged over 2009-2018, as data for 2007-2008 was not available.
****-** Formed on March 1, 2008 as a result of merging of Chita Oblast and Agin-Buryat Autonomous Okrug, after a referendum

Supplementary Table 12. Poisson regression analysis of risk factors for lip and oral cavity cancer (C00-C09) incidence, and oropharyngeal cancer (C10-C13) incidence (separately) for <u>both sexes</u> in the Russian Federation, 2008-2018. Bolded parameter estimates denote a negative association. Eleven variables were analysed in a backwards stepwise selection multivariate model. Coefficient ß indicates regression coefficient. I.R.R. denotes the incidence rate ratios. S.E. denotes standard error. CIs indicate confidence intervals. *p*-values <0.05 were considered significant. Only significant values are shown.

**Predictor**	**Coefficient ß**	**I.R.R.**	**CIs**	**S.E.**	***p*-value**
**Incidence Rate C00-C09 (Univariate Model)**					
Weekly alcohol consumption (2-3 times per week)	0.04	1.04	1.01-1.08	0.02	0.01
**Incidence Rate C00-C09 (Multivariate Model)**					
Weekly alcohol consumption (2-3 times per week)	0.05	1.05	1.01-1.08	0.02	0.007
**Incidence Rate C10-C13 (Univariate Model)**					
Weekly alcohol consumption (2-3 times per week)	0.05	1.05	1.00-1.11	0.03	0.05
Daily smoking	**-0.02**	0.98	0.96-1.00	0.009	0.02
Tuberculosis infection	**-0.01**	0.99	0.99-1.00	0.002	0.01
Syphilis infection	**-0.01**	0.99	0.98-1.00	0.004	0.03
**Incidence Rate C10-C13 (Multivariate Model)**					

Weekly alcohol consumption (2-3 times per week)	0.05	1.05	1.00-1.11	0.03	0.05

Supplementary Table 13. Poisson regression analysis of risk factors for lip and oral cavity cancer (C00-C09) incidence and oropharyngeal cancers (C10-C13) incidence (separately) for males in the Russian Federation, 2008-2018. Bolded parameter estimates denote a negative association. Eleven variables were analysed in a backwards stepwise selection multivariate model. Coefficient ß indicates regression coefficient. I.R.R. denotes the incidence rate ratios. S.E. denotes standard error. CIs indicate confidence intervals. *p*-values <0.05 were considered significant. Only significant values are shown.

**Predictor**	**Coefficient ß**	**I.R.R.**	**CIs**	**S.E.**	***p*-value**
**Incidence Rate Males C00-C09 (Univariate Model)**					
Rare alcohol consumption	**-0.01**	0.99	0.98-1.00	0.004	0.04
Weekly alcohol consumption (once per week)	0.02	1.02	1.00-1.03	0.01	0.008
Weekly alcohol consumption (2-3 times/week)	0.06	1.06	1.03-1.09	0.01	<0.00005
Smoking (<10 cigarettes/day)	**-0.01**	0.99	0.98-1.00	0.005	0.01
Smoking (>1 pack/day)	0.02	1.02	1.00-1.03	0.007	0.04
Gonorrhea infection	**-0.003**	1.00	0.99-1.00	0.002	0.03
**Incidence Rate Males C00-C09 (Multivariate Model)**					
Weekly alcohol consumption (2-3 times/week)	0.06	1.06	1.03-1.10	0.01	<0.00005
**Incidence Rate Males C10-C13 (Univariate Model)**					
Monthly alcohol consumption	**-0.03**	0.97	0.95-0.98	0.01	<0.0005
Weekly alcohol consumption (once per week)	0.03	1.03	1.01-1.05	0.009	0.001
Weekly alcohol consumption (2-3 times per week)	0.08	1.08	1.04-1.12	0.02	<0.00005
Never smoking	0.01	1.01	1.00-1.02	0.005	0.04
Rare smoking	0.06	1.06	1.02-1.10	0.02	0.003
Daily smoking	**-0.02**	0.98	0.96-0.99	0.007	<0.0005
Smoking (<5 cigarettes/day)	0.02	1.02	1.01-1.03	0.006	<0.0005
Smoking (>1 pack/day)	**-0.03**	0.97	0.95-1.00	0.01	0.04
Tuberculosis infection	**-0.01**	0.99	0.988-0.995	0.002	<1E-6
HIV infection	**-0.002**	1.00	0.996-1.00	0.001	0.03

Syphilis infection	**-0.01**	0.99	0.98-0.99	0.003	<0.0001
Gonorrhea infection	**-0.01**	0.99	0.986-0.995	0.002	<0.0001
Sale of alcohol content <9%	0.04	1.04	1.02-1.05	0.007	<1E-5
**Incidence Rate Males C10-C13 (Multivariate Model)**					
Any alcohol consumption	0.01	1.01	1.00-1.03	0.01	0.03
Weekly alcohol consumption (2-3 times per week)	0.08	1.08	1.02-1.14	0.03	0.006
Sale of alcohol content <9%	0.02	1.02	1.00-1.04	0.01	0.01
Alcohol induced psychosis and syndrome of dependence	0.01	1.01	1.00-1.02	0.004	0.04
Daily smoking	**-0.04**	0.96	0.93-0.98	0.01	0.0007
TB infection	**-0.004**	1.00	0.99-1.00	0.002	0.04

Supplementary Figure 1. Incidence of lip and oral cavity cancers (OCCs) (C00-C09) for males in the Russian Federation, 2007-2018. Each data point indicates the age-standardized incidence rate for a given year. The solid line represents the line of best fit, and dotted lines show the corresponding upper and lower 95% Confidence Intervals (CI). Coefficient of determination (R^2). Statistical significance is expressed in p-values. Slope -0.075/100,000 individuals per year, R^2=0.63, p=0.002 *CI; confidence interval*

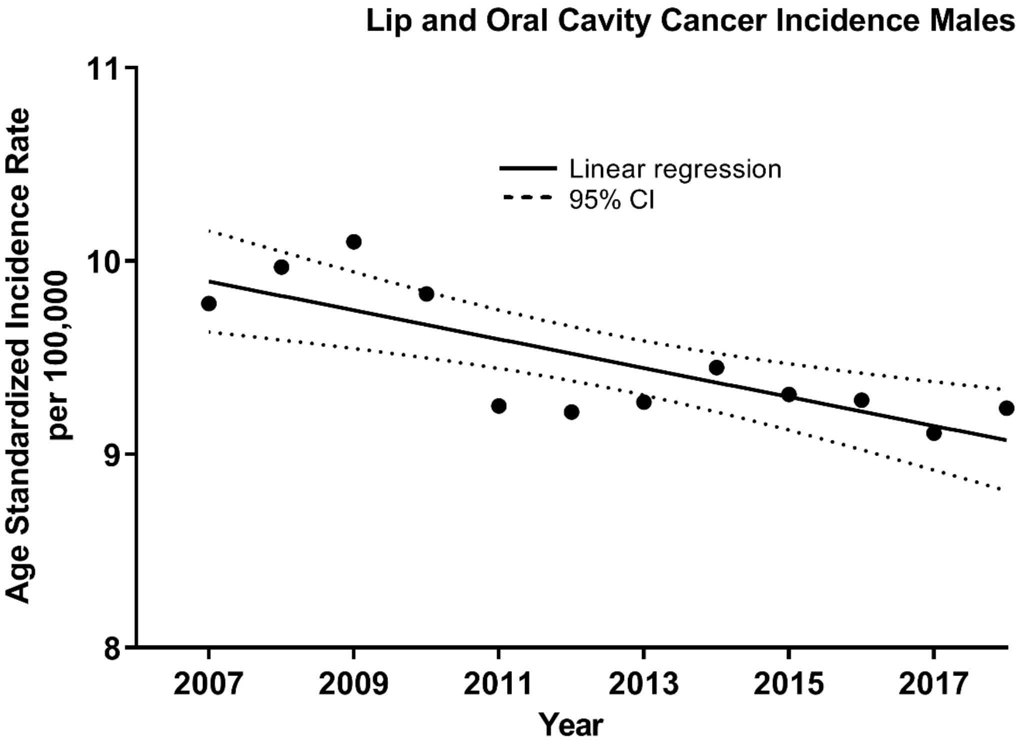

Supplementary Figure 2. Incidence of lip and oral cavity cancers (OCCs) (C00-C09) for females in the Russian Federation, 2007-2018. Each data point indicates the age-standardized incidence rate for a given year. The solid line represents the line of best fit, and dotted lines show the corresponding upper and lower 95% Confidence Intervals (CI). Coefficient of determination (R2). Statistical significance is expressed in p-values. Slope 0.036/100,000 individuals per year, R^2=0.65, p=0.0015 *CI; confidence interval*

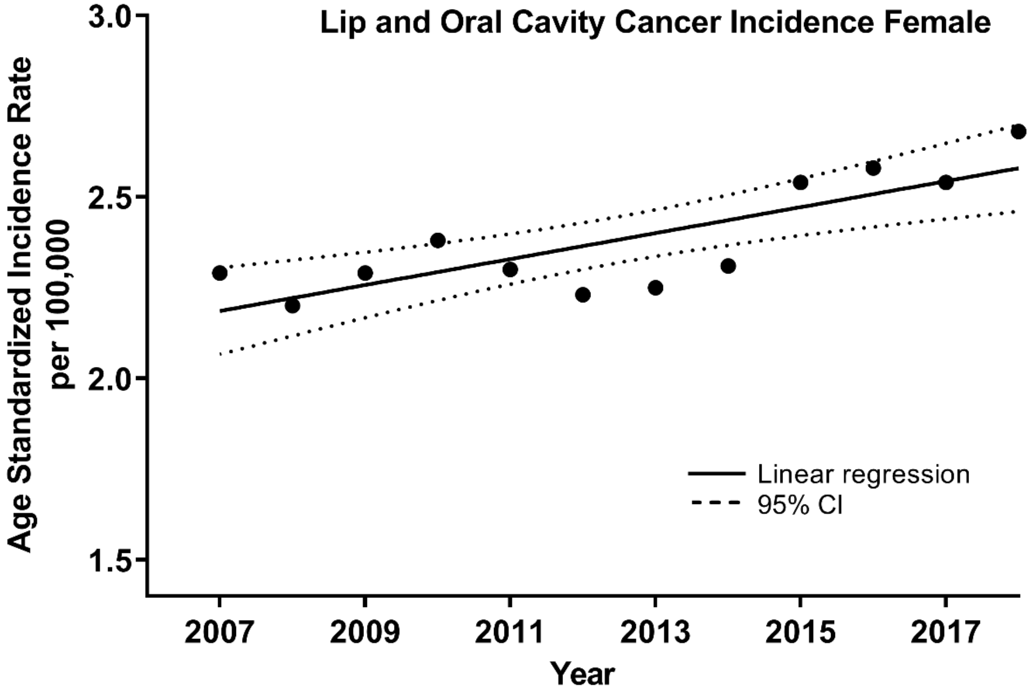

Supplementary Figure 3. Incidence rate ratio (IRR) of male-to-female lip and oral cavity cancers (OCCs) (C00-C09) in the Russian Federation, 2007-2018. A negative slope of -0.088 is observed, decreasing from 4.27 in 2007 to 3.45 in 2018 ($p<0.0001$). Coefficient of determination (R^2) = 0.85. Each data point indicates the age-standardized incidence rate for a given year. The solid line represents the line of best fit, and dotted lines show the corresponding upper and lower 95% Confidence Intervals (CI). Statistical significance is expressed in p-values. *CI; confidence interval*

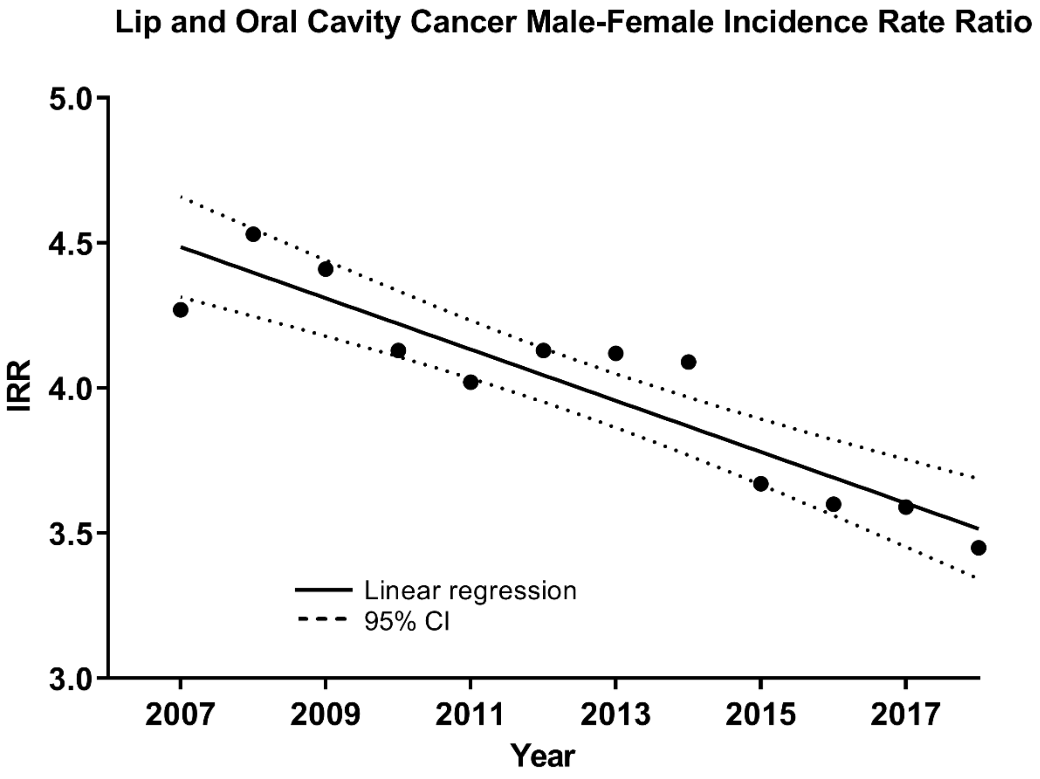

Supplementary Figure 4. Incidence of oropharyngeal cancers (OPCs) (C10-C13) for males in the Russian Federation, 2007-2018. Each data point indicates the age-standardized incidence rate for a given year. The solid line represents the line of best fit, and dotted lines show the corresponding upper and lower 95% Confidence Intervals (CI). Coefficient of determination (R^2). Statistical significance is expressed in p-values. Slope 0.048/100,000 individuals per year, R^2=0.53, p=0.007 *CI; confidence interval*

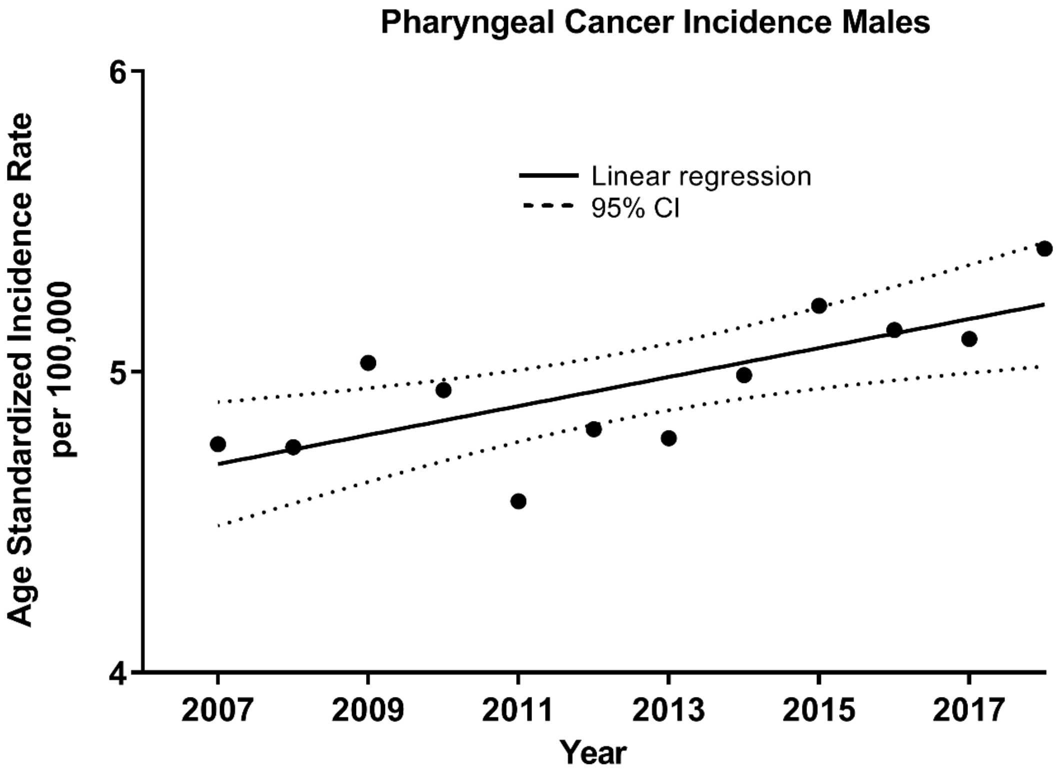

Supplementary Figure 5. Incidence of oropharyngeal cancers (OPCs) (C10-C13) for females in the Russian Federation, 2007-2018. Each data point indicates the age-standardized incidence rate for a given year. The solid line represents the line of best fit, and dotted lines show the corresponding upper and lower 95% Confidence Intervals (CI). Coefficient of determination (R^2). Statistical significance is expressed in p-values. Slope 0.014/100,000 individuals per year, R^2=0.67, p=0.001 *CI; confidence interval*

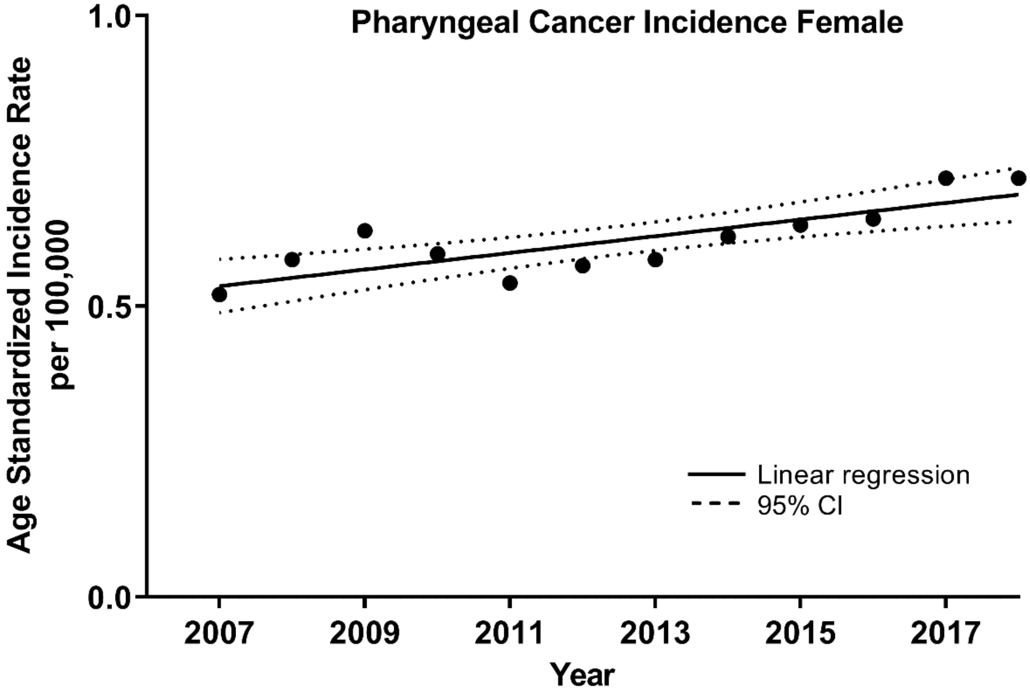

Supplementary Figure 6. Incidence rate ratio (IRR) of male-to-female oropharyngeal cancers (OPCs) (C10-C13) in the Russian Federation, 2007-2018. A negative slope of -0.10 is observed, decreasing from 9.15 in 2007 to 7.51 in 2018 (*p*=0.0038). Coefficient of determination (R^2) = 0.58. Each data point indicates the age-standardized incidence rate for a given year. The solid line represents the line of best fit, and dotted lines show the corresponding upper and lower 95% Confidence Intervals (CI).

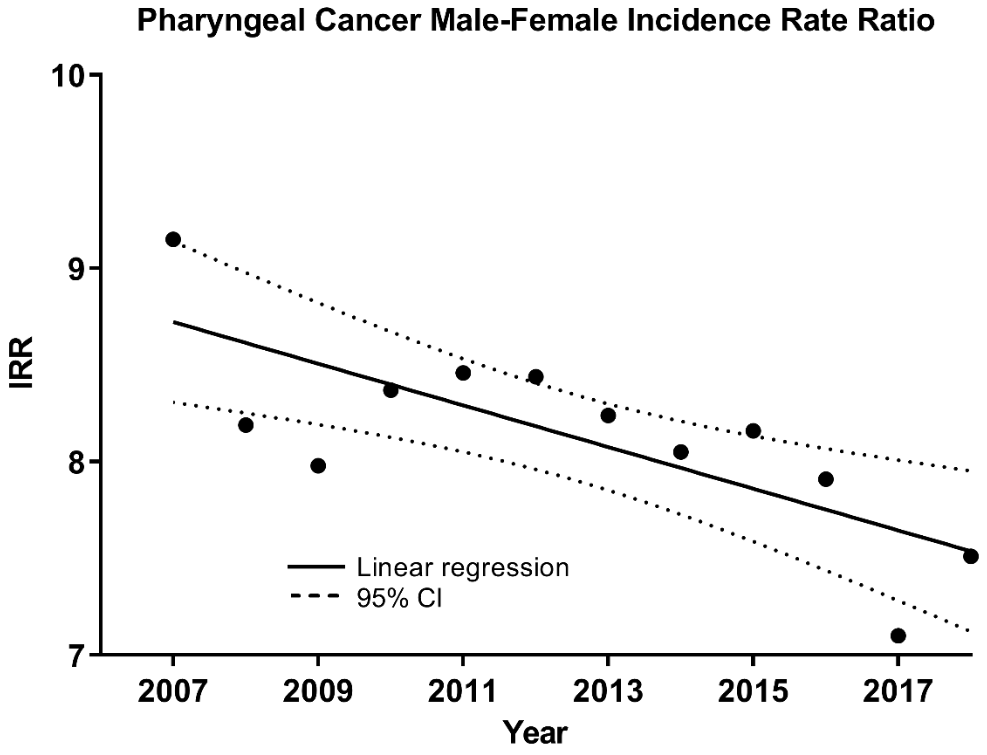

Supplementary Figure 7. Mortality of lip, oral cavity and oropharyngeal cancers (OCPCs) (C00-C14) for both sexes combined in the Russian Federation, 2007-2018. Each data point indicates the age-standardized incidence rate for a given year. The solid line represents the line of best fit, and dotted lines show the corresponding upper and lower 95% Confidence Intervals (CI). Coefficient of determination (R^2). Statistical significance is expressed in p-values. Slope 0.013/100,000 individuals per year, R^2=0.21, p=0.132 *CI; confidence interval*

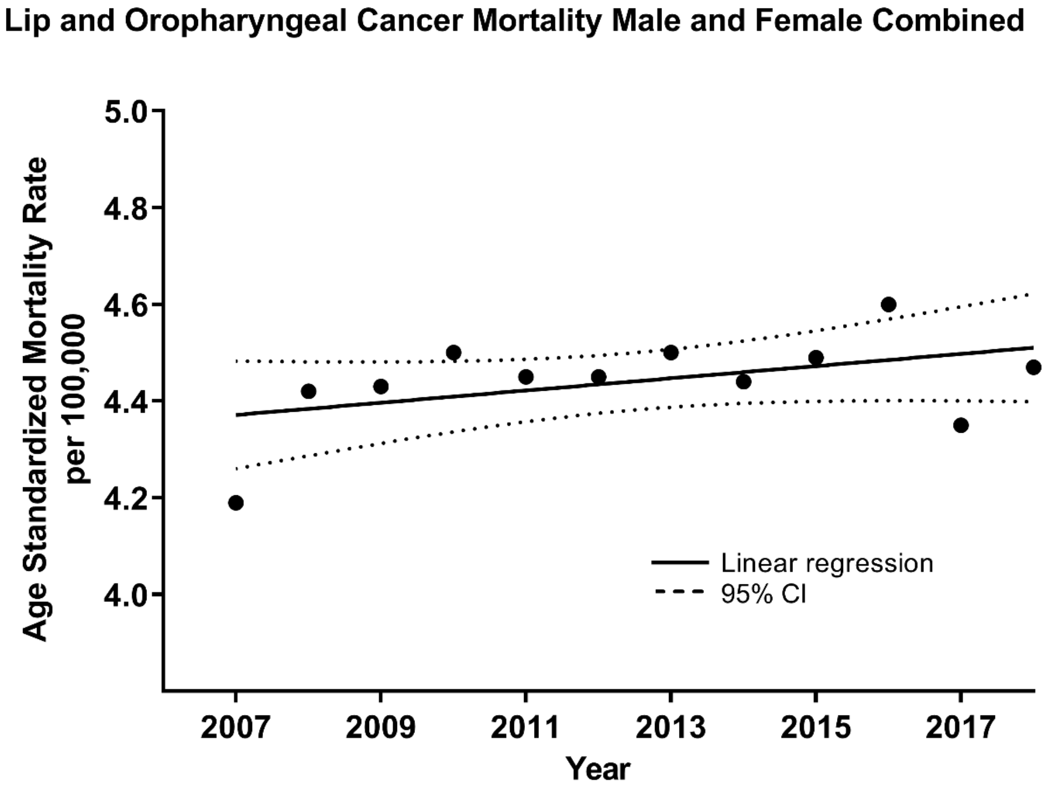

Supplementary Figure 8. Mortality of lip, oral cavity cancers and oropharyngeal cancers (OCCs and OPCs) (C00-C14) for males in the Russian Federation, 2007-2018. Each data point indicates the age-standardized incidence rate for a given year. The solid line represents the line of best fit, and dotted lines show the corresponding upper and lower 95% Confidence Intervals (CI). Coefficient of determination (R^2). Statistical significance is expressed in p-values. Slope -0.024/100,000 individuals per year, R^2=0.165, p=0.189 *CI; confidence interval*

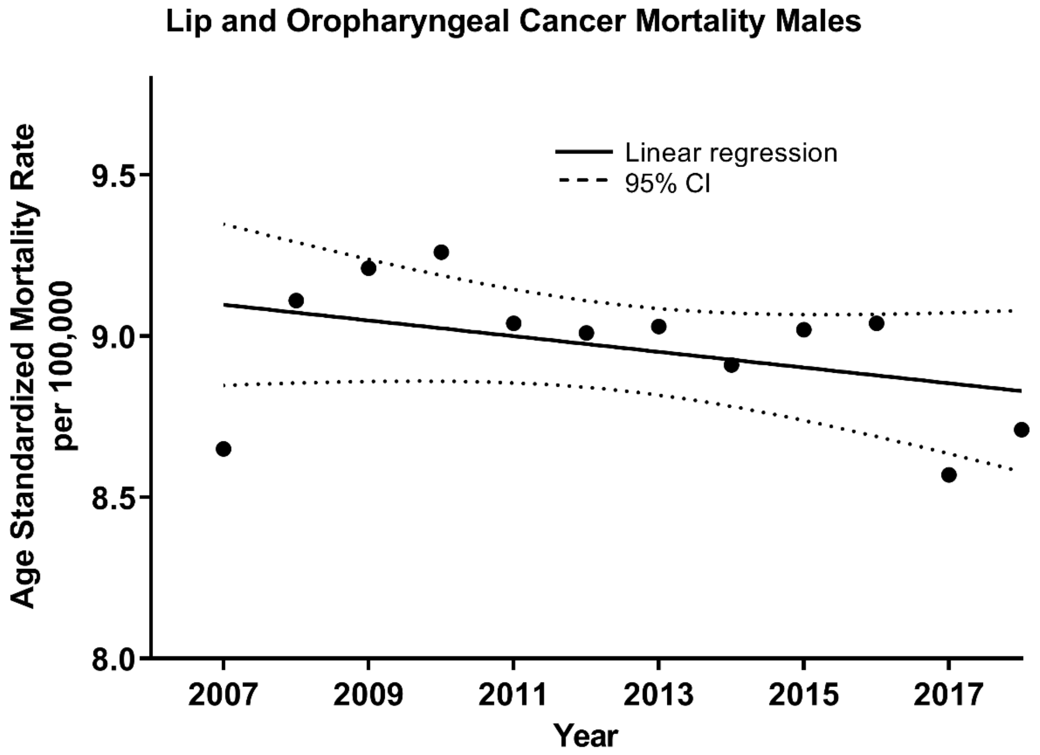

Supplementary Figure 9. Mortality of lip, oral cavity cancers and oropharyngeal cancers (OCCs and OPCs) (C00-C14) for females in the Russian Federation, 2007-2018. Each data point indicates the age-standardized incidence rate for a given year. The solid line represents the line of best fit, and dotted lines show the corresponding upper and lower 95% Confidence Intervals (CI). Coefficient of determination (R2). Statistical significance is expressed in p-values. Slope 0.023/100,000 individuals per year, R2=0.77, p=0.0002 *CI; confidence interval*

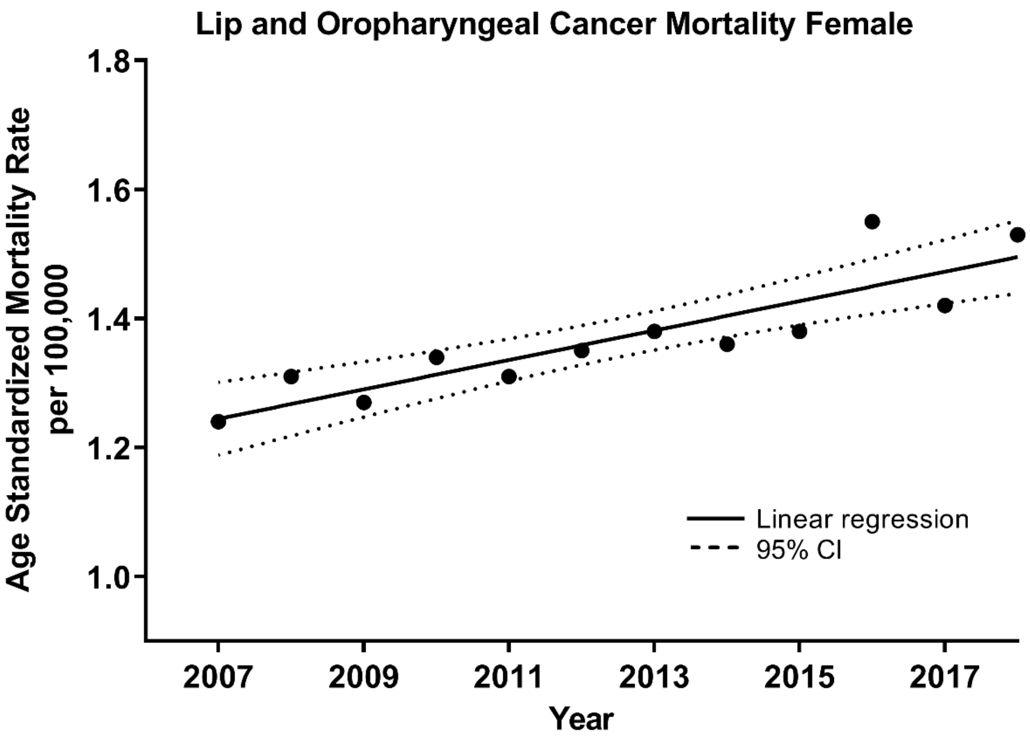

Supplementary Figure 10. Mortality rate ratio (MRR) male-to-female for lip, oral cavity cancers and oropharyngeal cancers (OCCs and OPCs) (C00-C14) in the Russian Federation, 2007-2018. A negative slope of -0.12 is observed, decreasing from 6.98 in 2007 to 5.69 in 2018 (*p*<0.0001). Coefficient of determination (R2) = 0.84. Each data point indicates the age-standardized incidence rate for a given year. The solid line represents the line of best fit, and dotted lines show the corresponding upper and lower 95% Confidence Intervals (CI).

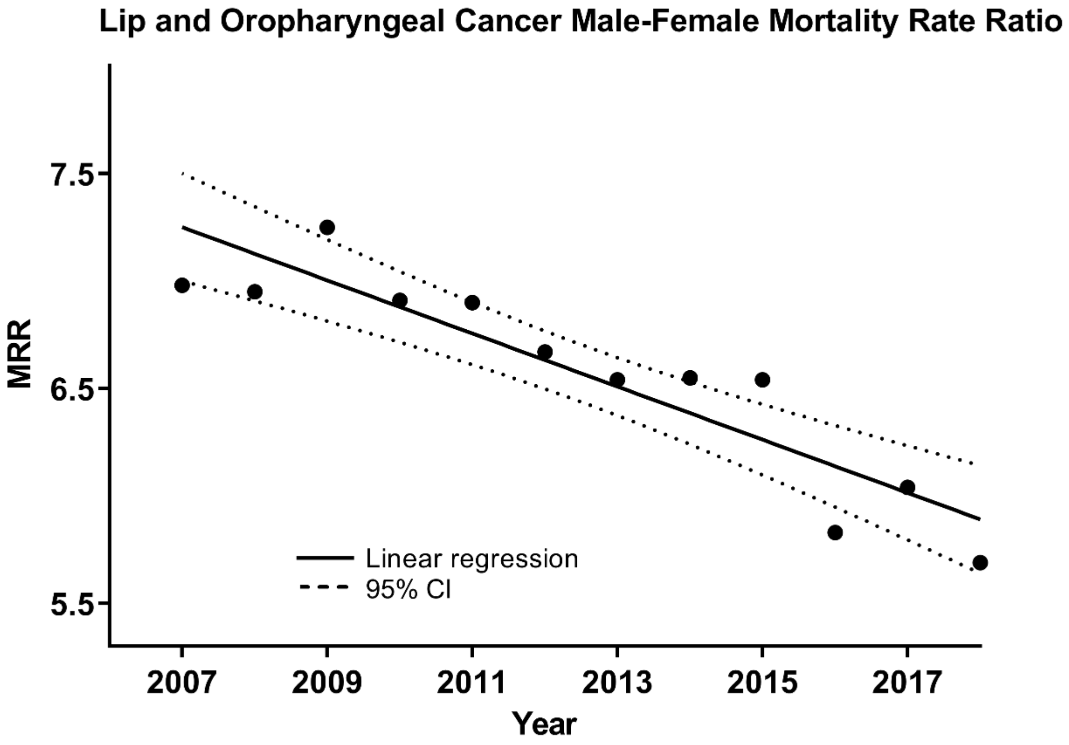

Supplementary Figure 11. Geographic distribution of lip and oropharyngeal cancers combined (C00-14) average age-standardized incidence rate for both sexes in the Russian Federation, 2008-2018.

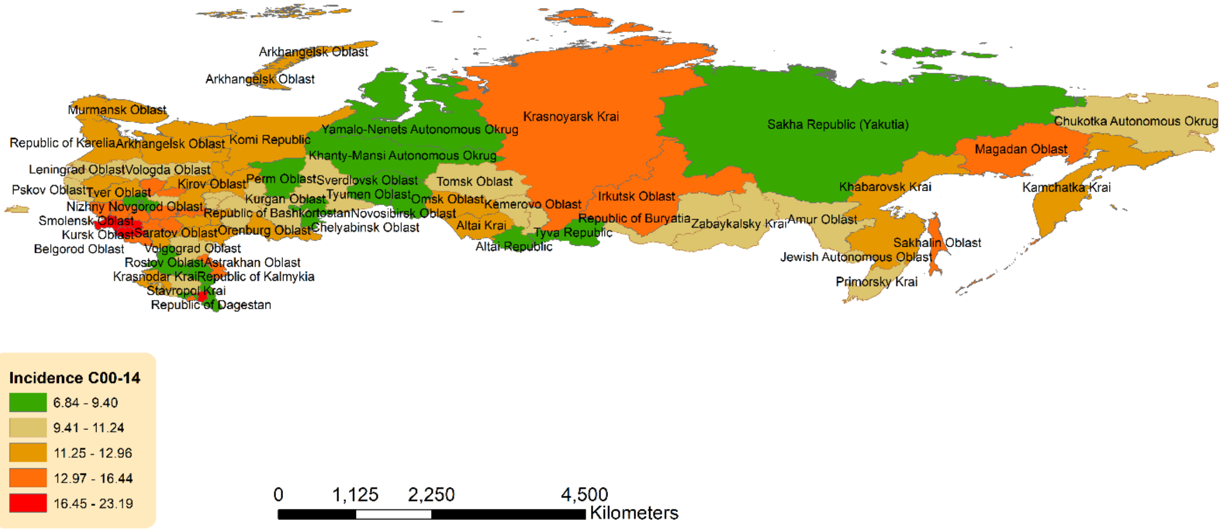

Supplementary Figure 12. Geographic distribution of lip, oral cavity and oropharyngeal cancer (OCCs and OPCs) (C00-14) average age-standardized mortality rate for both sexes combined in the Russian Federation, 2008-2018.

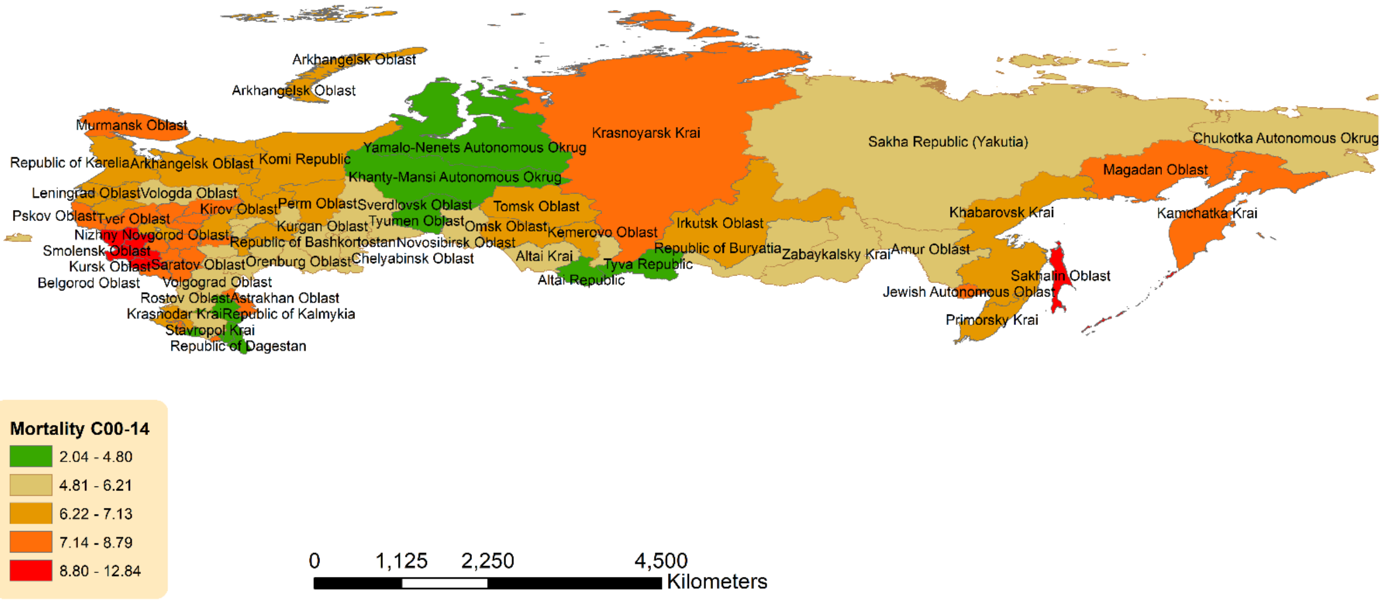

Supplement: Supplementary file 1 [file DataSheet_1.pdf]
